# Panel-Wide Screening of Tumour Cells of Diverse Histogenesis for Responsiveness to Silencing of miR-21, miR-17, and miR-155 by Mesyl Phosphoramidate Antisense Oligonucleotides

**DOI:** 10.3390/ijms27125446

**Published:** 2026-06-16

**Authors:** Svetlana Miroshnichenko, Olga Patutina, Olga Almieva, Ekaterina Burakova, Mikhail Maslov, Alesya Fokina, Dmitry Stetsenko, Marina Zenkova

**Affiliations:** 1 Institute of Chemical Biology and Fundamental Medicine SB RAS, Novosibirsk 630090, Russia; sveta-mira@yandex.ru (S.M.); patutina@1bio.ru (O.P.); yakovenko0lya@yandex.ru (O.A.); 2 Department of Physics, Novosibirsk State University, Novosibirsk 630090, Russia; ekaanabur@yandex.ru (E.B.); a.fokina@nsu.ru (A.F.); d.stetsenko@nsu.ru (D.S.); 3 Institute of Cytology and Genetics SB RAS, Novosibirsk 630090, Russia; 4 Lomonosov Institute of Fine Chemical Technologies, MIREA—Russian Technological University, Moscow 119571, Russia; mamaslov@mail.ru

**Keywords:** cancer, epithelial tumour cells, neuronal tumour cells, lymphoid tumour cells, antisense oligonucleotides, anti-miRs, miR-21, miR-17, miR-155, mesyl phosphoramidate

## Abstract

Molecular heterogeneity of malignant tumours remains a central challenge in oncology. Although neoplasms of distinct histogenesis share core oncogenic properties, the signalling networks sustaining these phenotypes are tumour-specific, highlighting the need for regulatory-level therapeutics adaptable to distinct molecular contexts. Oncogenic microRNAs (miRNAs), among which miR-21, miR-17, and miR-155 are broadly overexpressed in epithelial, lymphoid, and glial-neuronal neoplasms, represent attractive targets for therapeutic intervention. MiRNA inhibition by antisense oligonucleotides has emerged as a promising therapeutic strategy, and mesyl (methanesulfonyl) phosphoramidate oligonucleotides (µ-AMOs) represent a next-generation class of anti-miRNA agents with superior nuclease resistance and biological activity. Here, we report a systematic screening of 23 tumour cell lines of diverse histogenesis for sensitivity to paired combinations of µ-AMOs targeting miR-21, miR-17, and miR-155. Epithelial-derived lines exhibited the highest responsiveness, while lymphoid cell lines demonstrated the lowest sensitivity. The efficacy of specific µ-AMO pairs was found to be cell type-specific: combinations containing miR-21-targeting µ-AMO were most effective in epithelial lines, whereas the miR-17/miR-155-targeting pair showed superior activity in neuroglial models. Cellular responsiveness was driven by the extent of miRNA suppression, with no correlation detected for delivery efficiency or basal miRNA levels. The obtained results provide a basis for tumour-type-specific selection of µ-AMO combinations for clinical translation.

## 1. Introduction

Cancer remains one of the most pressing global health challenges. Despite major advances in molecular diagnostics and the development of targeted therapy, biological heterogeneity continues to pose a significant challenge to the effectiveness of anticancer therapies [1]. Although tumours from different tissues share core hallmarks of malignancy, including sustained proliferation, apoptosis resistance, invasiveness, and immune evasion, the molecular circuits supporting these phenotypes are highly context-dependent [2,3,4,5,6,7], reinforcing the need for therapeutics that act at the regulatory level to disrupt multiple oncogenic pathways simultaneously. Against this background, microRNAs (miRNAs) represent particularly attractive candidates. These short non-coding RNAs govern virtually all fundamental cellular processes through broad post-transcriptional networks in which each miRNA modulates numerous mRNA targets involved in cell cycle progression, evasion of apoptosis, and metabolic reprogramming [8,9,10]. As a consequence, dysregulation of miRNA expression upon cancer development profoundly perturbs these core homeostatic pathways, promoting malignant transformation, tumour progression, and metastatic dissemination.

Particular attention has been drawn to several highly oncogenic miRNAs, namely, miR-21, miR-17, and miR-155, which are among the best-characterised oncogenic drivers frequently implicated in epithelial, lymphoid, and glial-neuronal neoplasms [11,12,13]. A key challenge, however, is that the same miRNA may engage distinct signalling contexts depending on tumour histogenesis [14]. For instance, miR-21 operates through PI3K/Akt/mTOR signalling in epithelial and glial-neuronal tumours, but is more closely linked to NF-κB-driven programmes in lymphoid malignancies [15,16,17,18,19,20,21,22,23,24]. Similarly, miR-17 regulates cell-cycle and survival signalling, engaging PI3K/Akt and TGF-β pathways in epithelial cancers, mitochondrial apoptosis control and B-cell receptor signalling in lymphoid neoplasms, and a broader network including VEGF signalling, as well as PTEN and Bim-mediated apoptotic pathways in gliomas [25,26,27,28,29,30,31,32,33,34]. MiR-155 shifts from regulation of proliferative and invasive programs in epithelial tumours to cytokine and immune checkpoint signalling in lymphoid malignancies and survival pathways in glial-neuronal cancers [21,23,35,36,37,38,39,40,41,42,43,44,45,46,47,48,49,50,51,52,53,54]. Moreover, in certain molecular contexts, miRNAs may partially function as tumour suppressors. Together, these observations indicate that while miR-21, miR-17, and miR-155 are predominantly associated with oncogenic activity, their functional output is determined by tumour lineage and cellular signalling context.

Suppression of oncogenic miRNAs using chemically modified antisense oligonucleotides has emerged as a therapeutically recognised strategy [55,56]. In oncology, cobomarsen (MRG-106; miRagen/Viridian Therapeutics), an LNA-based antimiR-155, was evaluated in Phase I/II trials in mycosis fungoides, cutaneous T-cell lymphoma, CLL, and adult T-cell leukaemia/lymphoma, demonstrating an acceptable safety profile, although development was subsequently discontinued for reasons unrelated to safety or efficacy [57,58]. Additionally, anti-miR-221 (RGLS-221; Regulus Therapeutics) completed Phase I studies in solid tumours, including hepatocellular carcinoma and triple-negative breast cancer, with an acceptable safety and tolerability profile [59]. These examples establish proof of concept for the antisense inhibition of oncogenic miRNAs in vivo and highlight the importance of chemical optimisation of the oligonucleotide backbone for pharmacokinetic performance and therapeutic index. Among the modifications developed to date, mesyl (methanesulfonyl) phosphoramidate (µ or MsPA) internucleotidic linkage represents a particularly promising platform for next-generation anti-miR design, since it provides advantageous properties compared to classical phosphorothioate backbones [60].

**Figure 1:**
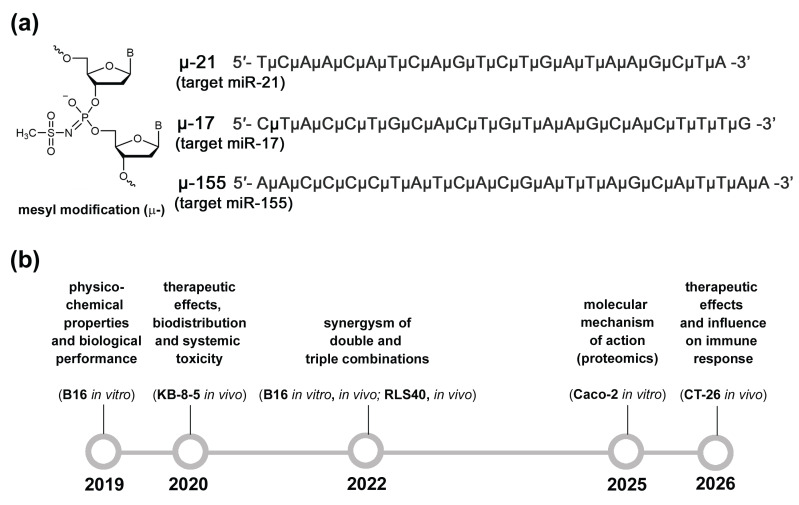

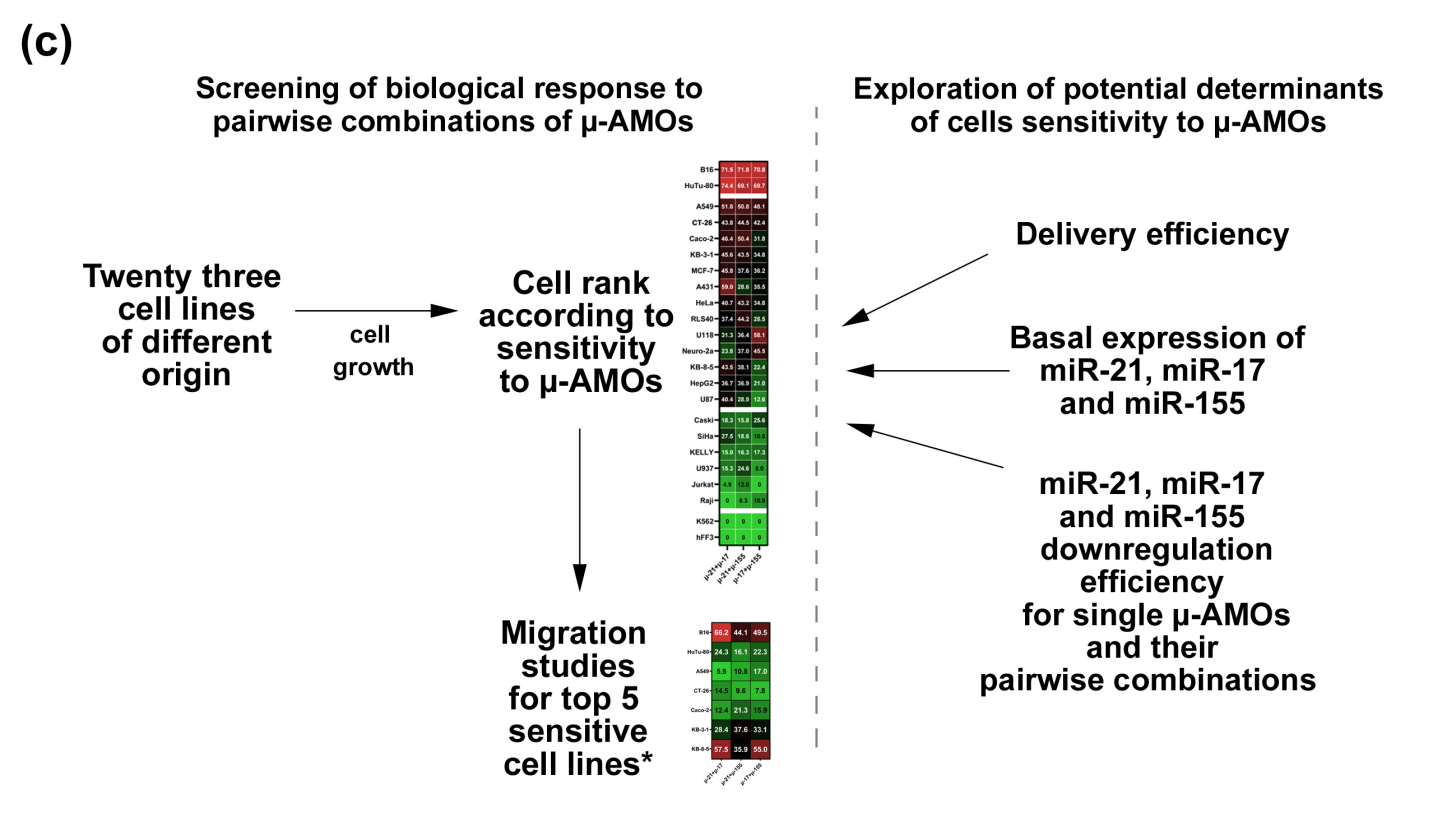
Current state of investigations related to µ-AMOs targeting miR-21, miR-17, and miR-155. (**a**) Structure of the mesyl modification of internucleotidic bonds and sequences of µ-AMOs investigated in the study. (**b**) Timeline of previous studies of µ-AMOs: biological performance in vitro and in vivo in several tumour models [60,61,62,63,64]. (**c**) Pipeline of the current screening of pairwise combinations of µ-AMOs. *—Migration studies were conducted on the top 5 sensitive cell lines as well as two cell lines with moderate sensitivity to µ-AMOs: KB-3-1 and KB-8-5.

We have been systematically investigating antisense oligonucleotides that substitute mesyl phosphoramidate groups for all internucleotidic phosphates (µ-AMOs) for targeting miR-17, miR-21, and miR-155 (Figure 1a). Over the past several years, we have made substantial progress in understanding both the mechanistic and biological properties of these compounds (Figure 1b). Our work has demonstrated that µ-AMOs possess sufficient hybridisation efficiency and specificity alongside remarkable resistance to serum nucleases, as well as the ability to recruit RNase H. Furthermore, µ-AMOs achieve efficient suppression of target miRNAs, significantly outperforming widely used phosphorothioate (PS) analogues across all measured parameters [60]. Across several cell lines, µ-AMOs targeting miR-21, miR-17, and miR-155 exhibited pronounced anti-proliferative, anti-migratory, and pro-apoptotic effects. Moreover, their application in paired and triple combinations resulted in a synergistic enhancement of these effects [60,61,62]. We also performed proteomic analysis of Caco-2 cells following µ-AMOs transfection, which enabled us to identify differential protein expression profiles and characterise global functional changes associated with the inhibition of miR-21, miR-17, and miR-155, caused either by the treatment with individual miRNA-specific µ-AMOs or by their triple combination [63]. Finally, evaluation of the therapeutic potential of µ-AMOs in tumour models (KB-8-5, B16, CT-26, and RLS_40_) demonstrated their high efficacy in suppressing tumour growth and metastasis (Figure 1b) [60,61,62,63,64]. Despite this substantial body of knowledge on µ-21, µ-17, and µ-155, their biological activity has so far been assessed in a limited number of models. A major unresolved question therefore remained: which types of tumours are the most sensitive to inhibition of miR-17, miR-21, and miR-155 by µ-AMOs. To address this, we performed a panel-wide evaluation of µ-AMO activity across 23 tumour cell lines of diverse origin (epithelial, lymphoid, neuronal, or glial) derived from both human and mouse sources (Table S1). Here, we present the results of the systematic screening, identifying tumour types most sensitive to paired inhibition of miR-21, miR-17, and miR-155 by µ-AMOs and delineating the key determinants of cellular responsiveness, including basal miRNA expression levels, transfection efficiency, and the extent of miRNA suppression. The obtained data identify the most responsive tumour types and establish a basis for the selection of µ-AMOs combinations for their prioritised treatment in future preclinical development.

## 2. Results

### 2.1. Pipeline of Current Investigation

Conceptually, the experimental design of the study consisted of two major parts. The first one focused on the assessment of biological activity. The central part of the study involved treatment of each cell line with pairwise µ-AMOs combinations (µ-17 + µ-21, µ-21 + µ-155, and µ-17 + µ-155), measurement of their effects on cell growth and ranking of the cell lines according to their sensitivity to µ-AMOs treatment. For the most responsive cell lines, we additionally assessed migration capacity under µ-AMO treatment using scratch assays or real-time monitoring with the xCELLigence system (Figure 1c).

It should be noted that in the present study, preference was given to a combined treatment strategy. Earlier, we demonstrated a synergistic enhancement of the antitumour effects of µ-AMOs when applied simultaneously in vitro and in vivo [62]. Here, we propose that simultaneous treatment of tumour cells with pairs of miRNA-targeted oligonucleotides will enable the concurrent inhibition of two oncogenic miRNAs, thereby affecting a broader range of molecular targets and signalling pathways. It may also help address tumour heterogeneity, as targeting two miRNAs increases the likelihood of suppressing diverse tumour cell subpopulations and reducing compensatory mechanisms that allow cells to adapt to anti-miRNA therapy. It should be stressed that the scope of the present study did not include the evaluation of interactions between μ-AMOs in combinatorial treatments. Therefore, the observed effects do not allow a direct assessment of whether the tested compounds act synergistically or additively.

The second part of the study focused on the analysis of determinants of sensitivity. To determine the potential reasons underlying more significant cellular response to µ-AMOs, we examined: (1) the efficiency of AMO delivery by cationic liposomes (2X3-DOPE), (2) basal levels of target miRNAs in the studied cell lines, and (3) the efficiency of miRNA downregulation by individual and pairwise combinations of µ-AMOs in cell lines with different sensitivity profiles.

Together, this two-stage approach provides a systematic framework for understanding cell-type-specific responses to the inhibition of miR-21, miR-17, and miR-155 by mesyl phosphoramidate antisense oligonucleotides.

### 2.2. Experimental Design

This study was performed across a panel of twenty-three cell lines with diverse histogenetic origins, including twelve (adeno)carcinomas of internal organs (epithelial origin), five leukaemia and lymphoma lines (lymphoid origin), four neuroblastoma and glioblastoma lines (neuronal–glial origin), one melanocytic cell line and one non-transformed fibroblast line (Table S1).

Basically, two main lines of investigation were addressed: (1) assessment of the sensitivity of cell lines to inhibition of miR-21, miR-17, and miR-155 using paired µ-AMOs combinations (µ-21 + µ-17, µ-21 + µ-155, and µ-17 + µ-155), including evaluation of effects on cell viability, migration, and miRNA downregulation efficiency; (2) investigation of the efficiency of intracellular oligonucleotide accumulation and target miRNA suppression following treatment with individual µ-AMOs (µ-21, µ-17, and µ-155).

Prior to this investigation, optimal µ-AMO concentrations were defined based on prior dose-response analyses in B16, Caco-2, CT-26, and KB-8-5 cells [62,63,64,65] (Figure S1). In most cell lines, the antiproliferative effects of µ-21 and µ-17 reached a plateau at 50–70 nM, with only a marginal 10–15% increase observed at higher concentrations up to 120–150 nM (Figure S1). Since 60 nM corresponds to a concentration at or near the maximum activity for the majority of constructs tested, it was selected as the dose for each component in the paired combinations. For a single µ-AMO application, a total concentration of 120 nM was selected, as it corresponds to the maximum effect and maintains the same total oligonucleotide load as the paired combinations, ensuring a valid comparison, while further dose increase would not significantly enhance biological performance.

As a control compound, a 22-mer fully µ-modified oligonucleotide (µ-Scr) lacking homology to the mammalian genome was used in this study.

Transfection of µ-AMOs was performed using cationic liposomes 2X3-DOPE, previously validated as an efficient delivery platform in both cellular and tumour models [66]. Experiments were conducted under standardised conditions at an N/P ratio of 4:1 (where N denotes the number of nitrogen groups in the 2X3 lipid and P the number of phosphate groups in the oligonucleotide), consistent with the optimal ratio established for cell culture systems [66].

Experimental timepoints were selected based on our previous studies: 72 h for the proliferation assay, 24 or 48 h for migration studies, 4 h for oligonucleotide accumulation studies, and 48 h for miRNA downregulation investigation [63,64,65].

### 2.3. Effect of Pairwise Combinations of µ-AMOs on Tumour Cell Growth

**Figure 2:**
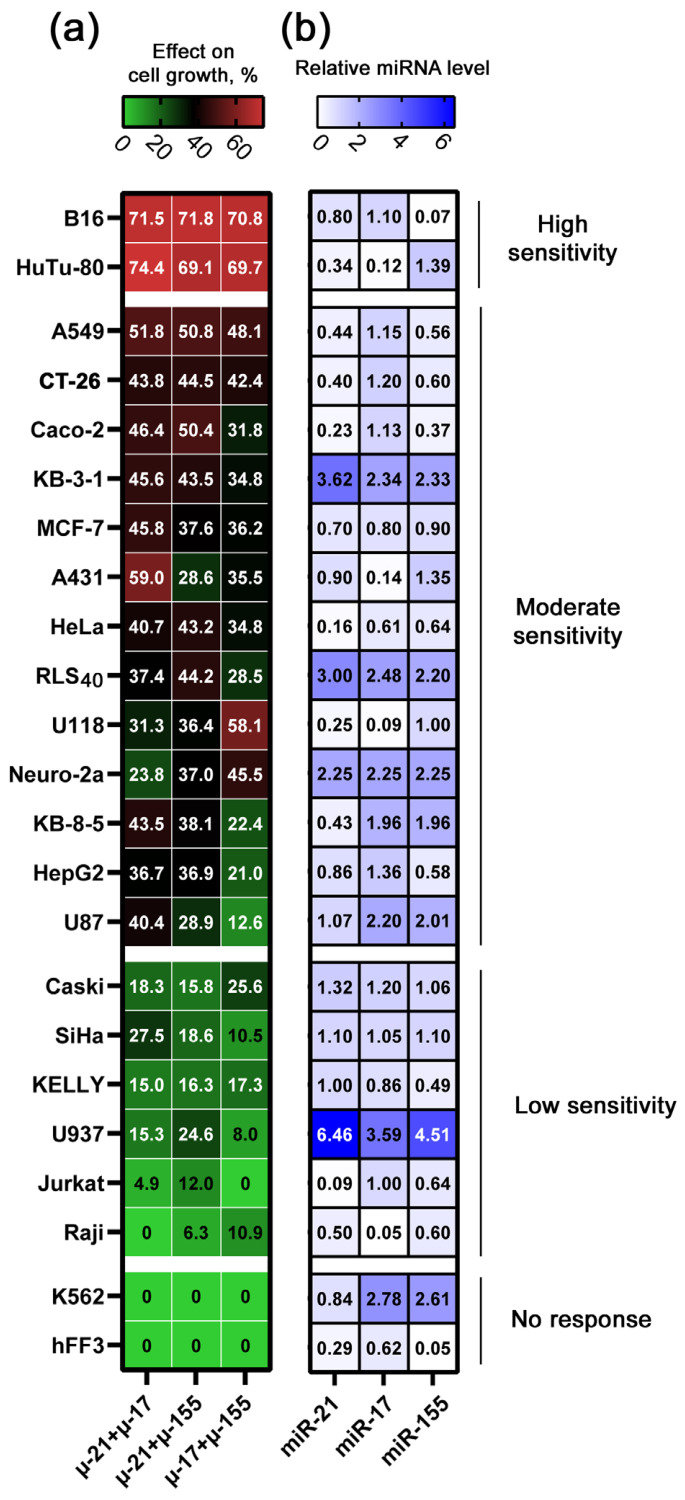
The effect of paired combinations of µ-AMOs targeting miR-21, miR-17, and miR-155 on tumour cell growth (**a**) and basal expression levels of these miRNAs in the corresponding cell lines (**b**). (**a**) Heatmap showing the effect of paired combinations µ-21 + µ-17, µ-21 + µ-155 and µ-17 + µ-155 on tumour cell growth. Results of the MTT test 72 h post-transfection with µ-AMOs targeting miR-21, miR-17, and miR-155 (60 nM of each, 120 nM in total) pre-complexed with cationic liposomes 2X3-DOPE. The data are represented as the mean effect (%) relative to the untreated control from at least three independent experiments. “High sensitivity”, “Moderate sensitivity”, “Low sensitivity” and “No response” categories demonstrate the sensitivity of the cells to combinatorial µ-AMOs treatment based on the effect of the compounds on cell growth. (**b**) Expression levels of miR-21, miR-17, and miR-155 in intact cells measured by stem-loop PCR. The level of miRNAs was normalised to the level of U6 RNA. Data are presented as the mean from at least three biological experiments and three technical replicates.

Cell growth was assessed under standardised conditions across all cell lines using the MTT/WST1 assay following cell transfection with µ-AMO pairs. This yielded a comprehensive dataset, which was visualised as a heat map representing cellular response to µ-AMO combinations (Figure 2a). Importantly, here and further in the manuscript, the term “cell growth” will be used when referring to the MTT assay results. This term is intended to encompass the potential effects of the studied compounds on cell proliferation, cell cycle regulation, apoptotic activity, and cellular metabolism.

Analysis of these data revealed that the studied cell lines can be divided into four groups according to their sensitivity to pairwise combinations of µ-AMOs, ranging from absolute non-responders to highly sensitive cells (Figure 2a). Thus, human chronic myeloid leukaemia cells K562 demonstrated a zero response to treatment with µ-AMO pairs. In these cells, none of the combinations µ-21 + µ-17, µ-21 + µ-155, or µ-17 + µ-155 exerted an anti-proliferative effect (Figure 2a, Table S2). Importantly, the absence of any effect of µ-AMO pairs was also observed in non-transformed human fibroblasts (hFF3). Low sensitivity to µ-AMO combinations was observed for Raji, Jurkat, U937, KELLY, Caski, and SiHa cells, for which the effect on tumour cell growth did not exceed 27% (Figure 2a, Table S2). Moderate sensitivity to µ-AMOs was demonstrated by A549, CT-26, Caco-2, KB-3-1, A431, MCF-7, RLS_40_, HeLa, U118, Neuro-2a, KB-8-5, HepG2, and U87 cell lines, for which a decrease in cell growth varied from 29 to 59% relative to the untreated control (Figure 2a, Table S2). The highest sensitivity to µ-AMOs was observed in human duodenal adenocarcinoma HuTu-80 and mouse melanoma B16 cells, for which the effect on tumour cell growth of all three combinations amounted to 69–74% (Figure 2a, Table S2).

So, the sensitivity of the studied cell lines to pairwise µ-AMO combinations increased in the following order: hFF3 and K562 << Raji < Jurkat < U937 < KELLY < SiHa < Caski < U87 < HepG2 < KB-8-5 < Neuro-2a < U118 < RLS_40_ < HeLa < A431 < MCF-7 < KB-3-1 < Caco-2 < CT-26 < A549 < HuTu-80 < B16.

A comparison of the activity of three µ-AMO combinations across the studied cell lines showed that the four most sensitive cell lines—B16, HuTu-80, A549, and CT-26—as well as those with moderate sensitivity—KB-3-1, MCF-7, and HeLa cells—exhibited similar sensitivity to all three µ-AMO combinations (Figure 2a, Table S2). It should be noted that these cells, with the exception of B16, are of epithelial origin.

It was observed that epithelial cell lines Caco-2, KB-8-5, and HepG2 were 1.5–2 times more sensitive to pairs containing µ-21 compared to the µ-17 + µ-155 combination (Figure 2a, Table S2). A similar pattern was observed for the epithelial cell line A431 and the glial cell line U87, where the µ-21 + µ-17 pair was up to three times more effective than the other combinations (Figure 2a, Table S2).

Finally, in Neuro-2a and U118 cell lines of neuroglial origin, the µ-17 + µ-155 combination showed up to 1.5 times higher inhibition of cell proliferation compared to the other ones (Figure 2a, Table S2).

Notably, the effect of cationic liposomes 2X3-DOPE on tumour cell growth did not exceed 5%, and the effect of control oligonucleotide μ-Scr varied from 1.5% to 20% across cell lines with different sensitivity to μ-AMOs (Table S3). Moreover, in cell lines exhibiting high or moderate sensitivity to μ-AMOs, even a twofold reduction in the dose of each oligonucleotide in pairs was sufficient to produce effects comparable to, or greater than, those observed for the corresponding single oligonucleotides (Table S4).

Overall, the lowest responsiveness to treatment with µ-AMO pairs was observed for lymphoid-derived cell lines, whereas tumour cells of epithelial origin and cell lines of melanocytic histogenesis demonstrated generally higher sensitivity to such treatment. Moreover, the relative efficacy of specific µ-AMO pairs depended on cellular origin: µ-21–containing combinations generally showed higher efficiency in epithelial cell lines, while µ-17 + µ-155 might represent the most effective combination for several neuronal-glial cell lines.

### 2.4. Evaluation of the Effects of Pairwise Combinations of µ-AMOs on the Migratory Potential of Tumour Cells

As a continuation of the study, we evaluated the effect of inhibition of miR-21, miR-17, and miR-155 by µ-AMO pairs on the migration activity of the five cell lines most sensitive to µ-AMO treatment according to cell growth assays: B16, HuTu-80, A549, CT-26 and Caco-2. In addition, two moderately sensitive cell lines of the same origin but differing in their multidrug resistance phenotype were examined: KB-3-1 and its multidrug-resistant subline KB-8-5. For the majority of cell lines, the effect of µ-AMOs on cell migration was assessed 48 h after cell transfection using the scratch assay. The xCELLigence real-time cell analysis system was used to evaluate the effects of μ-AMOs on the migratory potential of KB-3-1 and KB-8-5 cells, since they do not consistently form a uniform confluent monolayer, which limits the applicability of the scratch assay. The 48 h time point was selected to capture the maximal antimigratory effect in each cell line, as complete wound healing was observed across all groups at later time points (Figure S2). For HuTu-80 cells, a 24 h time point was used due to their higher migratory capacity.

**Figure 3:**
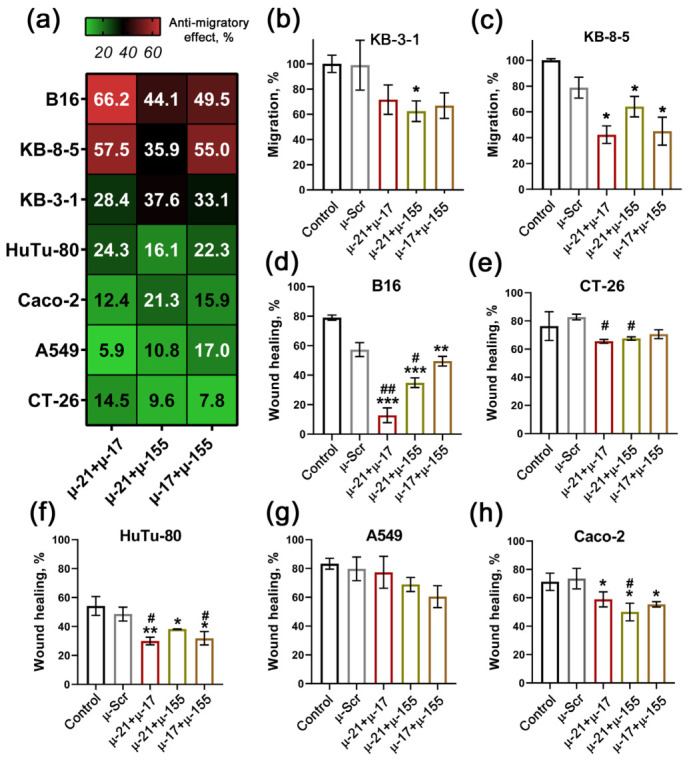
Anti-migrative effect of paired combinations of µ-AMOs targeting miR-21, miR-17, and miR-155. (**a**) Heatmap showing the effect of paired combinations µ-21 + µ-17, µ-21 + µ-155 and µ-17 + µ-155 on tumour cell migration 24–48 h post-transfection of cells with the respective µ-AMO pairs (60 nM of each, 120 nM in total) pre-complexed with cationic liposomes 2X3-DOPE. Results are represented as the mean effect in percentage relative to the Control. The anti-migratory effect of µ-AMOs on KB-3-1 (**b**) and KB-8-5 (**c**) cells was measured by the real-time registration system xCelligence RTCA; for B16 (**d**), CT-26 (**e**), HuTu-80 (**f**), A549 (**g**) and Caco-2 (**h**) cells, it was assessed by the wound-healing assay. Control—intact tumour cells. #, ##—statistically significant differences from the control oligonucleotide µ-Scr with *p* ˂ 0.05, and *p* ˂ 0.01, respectively. *, **, ***—statistically significant differences from the Control with *p* ˂ 0.05, *p* ˂ 0.01, and *p* ˂ 0.001, respectively. Data are presented as mean ± SEM from at least three biological experiments and three technical replicates.

Among the tested lines, the most pronounced anti-migratory effect of µ-AMOs was observed for B16, KB-8-5, and KB-3-1 cells (Figure 3a). In KB-3-1 cells, all three combinations produced comparable inhibition of tumour cell migration, amounting to approximately 28–37% relative to control and µ-Scr (Figure 3a,b). In KB-8-5 cells, the combination µ-21 + µ-155 reduced cell migration by 35.9% (13.9% vs. µ-Scr), whereas combinations containing µ-17 (µ-21 + µ-17 and µ-17 + µ-155) caused a stronger inhibition of migration by 55–58% (33–35% vs. µ-Scr) (Figure 3a,c). In B16 cells, pairs containing µ-155 (µ-21 + µ-155 and µ-17 + µ-155) decreased migration by 44–49% (22.4% vs. µ-Scr for µ-21 + µ-155), while the µ-21 + µ-17 pair produced the strongest effect, reducing migration by 66% (44.5% vs. µ-Scr) (Figure 3a).

A modest anti-migratory effect of µ-AMOs was observed in four cell lines identified as the most sensitive in cell viability assays. In CT-26 cells, µ-21-containing pairs (µ-21 + µ-17 and µ-21 + µ-155) reduced their migration by 10–14% (Figure 3a,b); in HuTu-80 cells, all three µ-AMO pairs produced a comparable inhibition amounting to 16–24% relative to control (Figure 3a,f). In contrast, in A549 and Caco-2 cells, the strongest anti-migratory effect—11–17% and 16–21%, respectively—was observed for µ-155-containing combinations (Figure 3a,g,h). For these cell lines, µ-Scr did not exhibit significant inhibition of tumour cell migration (Figure 3e–h).

We found that only in B16 melanoma cells do µ-AMO pairs exhibit an anti-migratory effect comparable in magnitude to their effect on cell viability. In contrast, in the four most sensitive cell lines—HuTu-80, A549, CT-26, and Caco-2—the reduction in migratory potential was markedly less pronounced than the decrease in cell viability. It can be assumed that the target miRNAs—miR-17, miR-21, and miR-155 in HuTu-80, A549, CT-26, and Caco-2 cells—are more strongly involved in the regulation of cell proliferation than migration. Notably, our recent proteomic analysis of Caco-2 cells demonstrated that transfection of these cells with µ-21, µ-17, and µ-155 significantly altered the expression of a broad range of proteins involved in mitotic spindle assembly, nuclear pore reorganisation during mitosis, and related processes [63]. It is likely that in KB-3-1 and KB-8-5 cells, miR-17, miR-21, and miR-155 play a more prominent role in regulating their migratory activity.

### 2.5. Investigation of Potential Determinants of Tumour Cell Sensitivity to µ-AMO Treatment

#### 2.5.1. Assessment of Intracellular Accumulation of AMOs Mediated by Cationic Liposomes 2X3-DOPE

One of the key factors that could significantly affect cellular response to anti-miRs is the efficiency of intracellular accumulation of the compounds in tumour cells. In this study, cationic 2X3-DOPE liposomes were used as the delivery vehicle [66]. Transfection efficiency was measured by flow cytometry as intracellular accumulation of a FAM-labelled oligonucleotide (150 nM) pre-complexed with 2X3-DOPE liposomes (N/P ratio 4/1) 4 h after addition of the lipoplexes to the cells (Table 1). The concentration of 150 nM was selected based on previously optimised conditions to ensure reliable fluorescent signal detection.

Under the experimental conditions used, 2X3-DOPE liposomes provided high transfection efficiency across all tested cell lines, regardless of histogenesis or responsiveness to miRNA-targeted µ-AMOs pairs. Specifically, 81–96% of the cell population was positive for oligonucleotide uptake in all studied cell lines (Table 1). Analysis of mean fluorescence intensity (RFU) revealed up to a 20-fold variability in the level of FAM-ON intracellular accumulation. The lowest RFU value (0.6 × 10^5^) was detected for A549 cells, which are among the top five most sensitive lines to µ-AMOs (Table 1). An intermediate level of accumulation (3–6 × 10^5^ RFU) was observed in several cell lines, including the highly sensitive B16 and HuTu-80, moderately sensitive HeLa and Neuro-2a, as well as the non-responder K562 cells (Table 1). The highest accumulation of the fluorescently labelled oligonucleotide (14–15.5 × 10^5^ RFU) was detected in Jurkat, Raji and hFF3 cells, classified as low sensitive and non-responding to µ-AMOs (Table 1).

Taken together, these findings suggest that, within the framework of this study, the 2X3-DOPE delivery system enables efficient oligonucleotide accumulation across cell lines of diverse histogenetic origin. These results further indicate that the efficiency of oligonucleotide accumulation is unlikely to be the primary factor determining the magnitude of the cellular response to miRNA-targeted μ-AMOs. Instead, the magnitude of the therapeutic response appears to be governed by cell line–specific molecular mechanisms.

#### 2.5.2. Analysis of Basal Expression Levels of miR-21, miR-17, and miR-155 in Cells of Different Histogenetic Origin

Differences in the intensity of cellular response to miRNA-targeted µ-AMO treatment may also be potentially attributed to the differences in the expression levels of these miRNAs in the cells. Basal expression levels of miR-21, miR-17, and miR-155 in the cells were determined using stem-loop RT-PCR (Figure 2b and Figure S3a–c).

Based on target miRNA expression, the analysed cell lines could be divided into two main groups. The first group included U937, KB-3-1, RLS_40_, Neuro-2a, K562, U87 and KB-8-5 cells, predominantly of lymphoid or neuronal-glial origin, which exhibited high basal levels of the target miRNAs—2–3.5-fold higher relative to the reference snRNA U6 (Figure S3a). The second group consisted mainly of epithelial cell lines, characterised by moderate or low basal expression levels of these miRNAs—equal to or 2.5–20-fold lower relative to U6 snRNA (Figure S3b,c).

When classified according to the expression pattern of the three target miRNAs, the cell lines could be further subdivided into three categories (Figure 2b). The first category comprised cells in which all three miRNAs were expressed at similarly high levels (KB-3-1, Neuro-2a, RLS_40_, MCF-7, Caski, and SiHa), predominantly of epithelial origin. The second category included cells in which the expression levels of two miRNAs exceeded that of the third one by 2–9.6-fold (B16, HeLa, U87, KELLY, Jurkat, Raji, KB-8-5, K562, and A431), belonging mainly to neuronal or lymphoid origin. The third category consisted of cell lines in which one miRNA was expressed at a level 2.5–12-fold higher than the other two miRNAs (A549, HuTu-80, CT-26, Caco-2, U118, HepG2, and hFF3), predominantly of epithelial origin.

Comparison of basal expression levels of individual miRNAs revealed that miR-21 expression was the lowest in Jurkat and HeLa cells and the highest in U937 cells (Figure 2b). A similar pattern was observed for miR-17, with low expression detected in HuTu-80, U118, Raji, and A431 cells and high expression in U937 and K562 cells (Figure 2b). The lowest miR-155 expression was detected in hFF3 and B16 cells, whereas the highest level was observed in U937 cells (Figure 2b).

Integration of these data did not reveal a statistically significant correlation between basal levels of miR-21, miR-17, and miR-155 and cellular sensitivity to miRNA-targeted μ-AMOs; however, the data suggested a possible trend where the highest expression of all three miRNAs was observed in U937 cells (lymphoid origin), and lowest expression of all three miRNAs was detected in Raji cells (also of lymphoid origin) and non-transformed fibroblasts hFF3, and these three lines were characterised by low or no sensitivity to µ-AMO treatment.

#### 2.5.3. Evaluation of Target miRNA Suppression Efficiency Induced by µ-AMO Treatment in Tumour Cells

**Figure 4:**
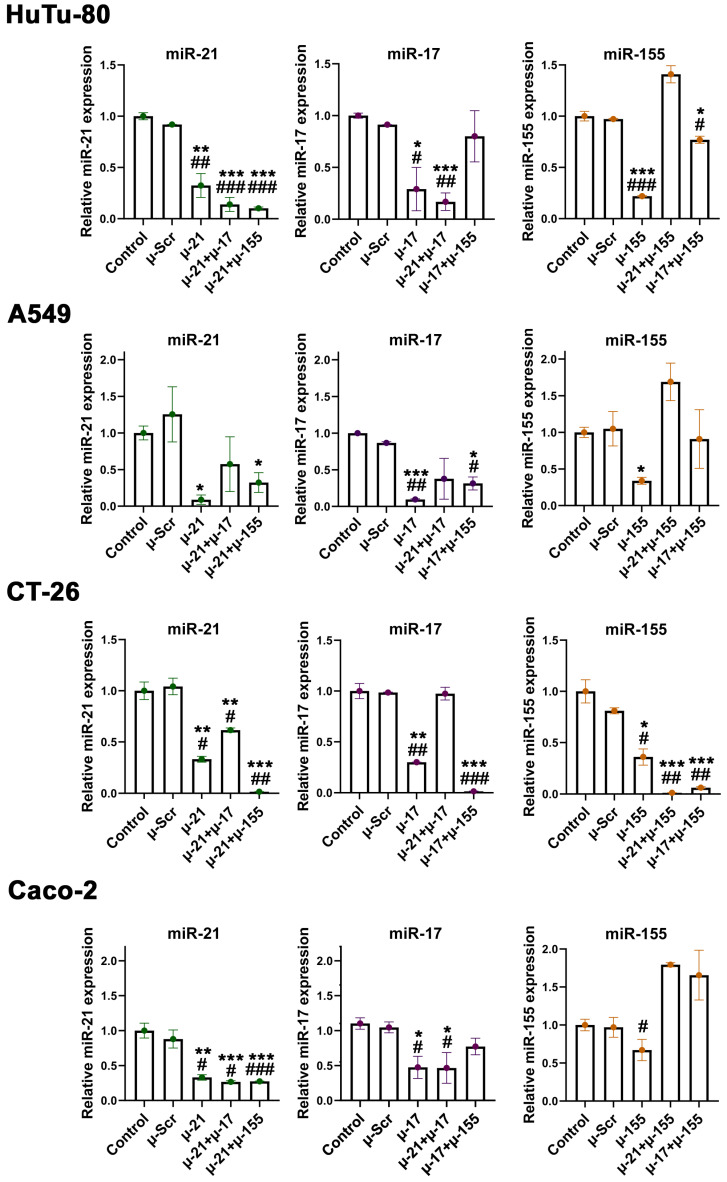
Expression levels of miR-21, miR-17, and miR-155 following cell treatment with individual or paired µ-AMOs in cell lines HuTu-80, A549, CT-26, and Caco-2. Relative expression of miR-21, miR-17, and miR-155 was measured by stem-loop PCR 48 h post-transfection with µ-AMOs in complex with 2X3-DOPE. The level of miRNAs was normalised to the level of U6 RNA. Control—intact tumour cells; µ-AMO (60 nM of each ASO in a pair, 120 nM for individually applied AMOs). *, **, ***—statistically significant differences from the Control with *p* ˂ 0.05, *p* ˂ 0.01, and *p* ˂ 0.001, respectively; #, ##, ###—statistically significant differences from µ-Scr with *p* ˂ 0.05, *p* ˂ 0.01, and *p* ˂ 0.001, respectively. Data are presented as mean ± SEM from at least three biological experiments and three technical replicates.

**Figure 5:**
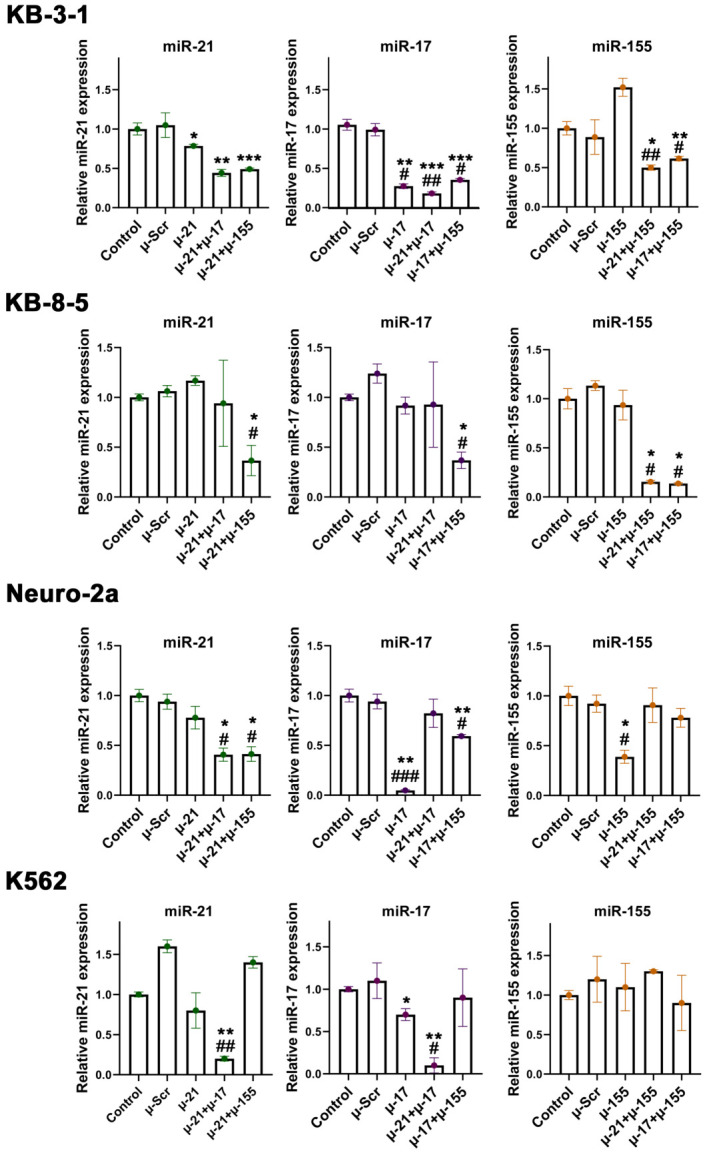
Expression levels of miR-21, miR-17, and miR-155 following treatment of moderately sensitive KB-3-1, KB-8-5, Neuro-2a, and non-responding K562 cells with individual or paired µ-AMOs. Relative expression of miR-21, miR-17, and miR-155 was measured by stem-loop PCR 48 h post-transfection with µ-AMOs in complex with 2X3-DOPE cationic liposomes. The level of miRNAs was normalised to the level of U6 RNA. Control—intact tumour cells; µ-AMO (60 nM of each AMO in a pair, 120 nM for individually applied AMOs). *, **, ***—statistically significant differences from the Control with *p* ˂ 0.05, *p* ˂ 0.01, and *p* ˂ 0.001, respectively; #, ##, ###—statistically significant differences from µ-Scr with *p* ˂ 0.05, *p* ˂ 0.01, and *p* ˂ 0.001, respectively. Data are presented as mean ± SEM from at least three biological experiments and three technical replicates.

We further analysed the efficiency of target miRNA silencing following cell treatment with µ-AMOs. The experiments were performed using cell lines classified as highly sensitive (HuTu-80) or moderately sensitive, including A549, Caco-2, KB-3-1 and KB-8-5, CT-26, Neuro-2a, as well as non-responding cells K562 (Figure 4 and Figure 5). miRNA levels were measured using stem-loop RT-PCR 48 h after cell transfection with µ-AMOs either as single oligonucleotides or in pairs pre-complexed with 2X3-DOPE.

The analysis demonstrated that the responsiveness of the cell lines to µ-AMOs treatment, evaluated by cell growth assay, is consistent with the efficiency of target miRNA suppression.

The most pronounced reduction in miR-21, miR-17, and miR-155 levels following transfection with the corresponding µ-AMOs was observed in the four most sensitive cell lines—HuTu-80, A549, CT-26, and Caco-2 (Figure 4). In HuTu-80 and CT-26 cells, individual µ-AMOs caused 66–78% inhibition of the miRNAs. In A549 cells, miR-21 and miR-17 were inhibited by 91% compared to the control and µ-Scr, whereas miR-155 suppression was less pronounced, averaging 66%. A similar pattern was observed in Caco-2 cells, where inhibition of miR-21 and miR-17 reached 57–65%, while miR-155 suppression was limited to approximately 33%.

In contrast, cell lines with reduced sensitivity (KB-3-1, KB-8-5, Neuro-2a, and K562) exhibited weaker and more variable effects of µ-AMOs used individually (Figure 5). In KB-8-5 cells, inhibition of miR-21, miR-17, and miR-155 did not exceed 8%, which may indicate different kinetics or regulatory dynamics of miRNA suppression in these cells. In K562 cells, no significant changes in miR-21 or miR-155 levels were detected following monotherapy, whereas the µ-AMO targeting miR-17 reduced its level by approximately 30%. In KB-3-1 cells, no inhibition of miR-155 was observed following treatment with µ-155 alone; however, miR-21 and miR-17 were reduced by 22% and 72%, respectively. In Neuro-2a cells, selective suppression was evident: miR-21, miR-17, and miR-155 levels decreased by 22, 91, and 61%, respectively, indicating a pronouncedly selective response pattern.

It should be noted that in the studied cell lines, the control oligonucleotide µ-Scr did not cause any significant downregulation of the target miRNAs (Figure 4 and Figure 5).

The transition from single µ-AMOs to pairwise combinations revealed distinct response patterns, which were also in agreement with the baseline sensitivity of cells to µ-AMOs.

In highly sensitive HuTu-80 and A549 cells, robust suppression of miR-21 and miR-17 was maintained across all double combinations, reaching up to 90% and matching or exceeding the effects of mono oligonucleotides (Figure 4). However, combinations containing µ-155 (µ-21 + µ-155 and µ-17 + µ-155) produced no more than 20% inhibition of miR-155 or no detectable effect.

In CT-26 cells, combinations containing µ-155 (µ-21 + µ-155 and µ-17 + µ-155) produced stronger miRNA suppression compared to monotherapy (99% vs. 67%), whereas other combinations showed effects comparable to single µ-AMOs. A similar trend was observed in KB-8-5 cells, where all combinations comprising µ-155 showed a greater effect than monotherapy (0–8% vs. 64–72%), while other µ-AMO pairs were less effective than the corresponding single µ-AMOs (Figure 5).

In Caco-2 cells, the effect of all pairwise combinations was equal to or lower than that observed with monotherapy, which may be directly related to the twofold reduction in the concentration of each specific oligonucleotide in the combined setting. In contrast, in KB-3-1 cells, all pairwise combinations resulted in higher miRNA suppression compared to monotherapy (22% vs. 51% for miR-21, 72% vs. 82% for miR-17, and 0% vs. 50% for miR-155).

In Neuro-2a cells, the effect of all combinations was generally weaker than that of monotherapy, except for combinations containing µ-21, where miR-21 suppression increased from 22% with single µ-21 to 59% with µ-21 + µ-17 and µ-21 + µ-155 (Figure 5).

In the non-responder K562 line, a measurable effect was observed only with the µ-21 + µ-17 combination, whereas other combinations had no significant impact on target miRNA levels, indicating a highly selective combinatorial response in this line (Figure 5).

Overall, the most effective suppression of miR-21, miR-17, and miR-155 was observed in the four most sensitive cell lines—HuTu-80, A549, CT-26, and Caco-2—whereas moderately sensitive lines (KB-3-1, KB-8-5, Neuro-2a) and non-responding K562 cells exhibited substantially weaker and more variable responses to µ-AMOs. The transition to pairwise combinations revealed cell-specific patterns of interaction, ranging from preserved or enhanced effects (HuTu-80, CT-26, KB-3-1) to attenuation or complete loss of activity (Caco-2, Neuro-2a). Notably, K562 cells, which did not respond to µ-AMOs in cell growth assays, displayed the weakest overall impact of these oligonucleotides on target miRNA levels among all studied lines.

## 3. Discussion

One of the major challenges in the development of anticancer therapeutics is the pronounced heterogeneity of malignant neoplasms. As a consequence, effective antitumour therapy increasingly requires not a single agent, but combinations of therapeutics capable of simultaneously targeting both phenotypically distinct neoplasms and heterogeneous cellular populations within a single tumour node. In this regard, the concept of miRNA-mediated regulation of carcinogenesis remains a highly relevant and promising field of research. Among the numerous miRNA regulators with broad targetomes, several universal and particularly potent therapeutic candidates can be identified, including miR-21, miR-17, and miR-155. Since the spectrum of targets controlled by these miRNAs may vary substantially across tumours of different histogenetic origins, evaluation of tumour sensitivity to compounds targeting these miRNAs represents a critical stage in drug development.

In the present study, we performed a large-scale screening of 23 tumour cell lines treated with paired combinations of mesyl phosphoramidate oligonucleotides targeting miR-21, miR-17, and miR-155. We demonstrated that the responsiveness of tumour cells of different histogenetic origins to treatment with µ-AMO pairs varies considerably, from no response to pronounced inhibition of tumour cell growth.

Since miR-21, miR-17, and miR-155 are deeply involved in the regulation of basic pro-oncogenic processes, during the screening, we focused on the effects of µ-AMO pairs on the growth rate and migratory activity of tumour cells of different histogenetic origins. We found that higher sensitivity to anti-miRNA therapy was demonstrated by melanocytic and epithelial-derived cells, especially for combinations containing the miR-21-targeting oligonucleotide. In addition, several neuronal–glial tumour cell lines displayed selective sensitivity to the oligonucleotide pairs targeting miR-17 and miR-155. In contrast, lymphoid-derived cells tended to exhibit weak or no response to µ-AMO treatment. We hypothesised that the observed sensitivity pattern may be explained by the especially broad targetome of miR-21 in tumours of epithelial origin, where the key targets include *PTEN*, *PDCD4*, *TPM1*, and *TRAF4* [15,16,17,18,19,20]. At the same time, oligonucleotides targeting miR-17 and miR-155 may complement the therapeutic spectrum of miR-21 by affecting additional differentially expressed targets involved in cell-cycle regulation and tumour progression, including the growth factor-associated protein *TGFBR2*, and other key proliferation- and apoptosis-related targets such as *PIK3R1*, caspase-3, and *TIMP2* for miR-17 [25,26,27,28,29], as well as apoptosis-associated proteins and signalling regulators such as *Jade-1*, *AIF*, *CDKN1B*/*p27Kip1*, *TP53INP1*, and *MAP3K10* for miR-155 [35,36,37,38,39,40,41,42,43,44,45,46,47,48]. As a result, coordinated modulation of the targetomes of two miRNAs may induce large-scale reorganisation of carcinogenesis-associated pathways, ultimately producing a pronounced effect on tumour cell viability.

A unique specific pattern was also evaluated for several neuronal–glial tumour models. The increased specificity of the µ-17 + µ-155 pair was observed in Neuro-2a and U118 cells, which may be associated with the extensive repertoire of miR-17-regulated targets in these tumours, including *PTEN*, *MAP3K5*, *PIK3CB*, *PRKACB*, *WEE1*, *CCND1*, *VEGFA*, *PTPRO*, *TP53INP1*, *BCL2L11*, and *E2F1* [30,31,32,33,34]. Simultaneously, miR-155 inhibition may promote these effects through modulation of TERF1-dependent pathways and suppression of *WDR82*, thereby affecting tumour-cell survival and resistance to programmed cell death [23,54].

Moreover, our results indicate that suppression of miR-21, miR-17, and miR-155 primarily affects tumour cell growth, thereby contributing substantially to the reduction of the malignant phenotype. This observation may be explained by the strong involvement of the target genes of these miRNAs in cell-cycle progression and regulation of cell growth [15,16,17,18,19,20,21,30,31,32,33,34]. In addition, our previously obtained proteomic data from Caco-2 cells transfected with µ-21, µ-17, and µ-155 oligonucleotides demonstrated that suppression of miR-21, miR-17, and miR-155 by specific oligonucleotides induces coordinated alterations of entire protein clusters associated with proliferation, cell-cycle regulation, and mitotic processes [63]. In contrast, only a limited number of differentially expressed proteins were associated with the regulation of tumour cell motility, and only a single cluster related to cell adhesion was observed in the case of µ-21-treated Caco-2 cells.

In searching for potential factors underlying the tissue-specific differences in responsiveness to µ-AMOs, we found that AMO intracellular accumulation is not the limiting determinant of cellular sensitivity to these compounds. Although lymphoid cells are generally known to be more difficult to transfect [67,68], our delivery platform based on cationic liposomes 2X3-DOPE enables highly efficient accumulation of AMOs in cell lines of different histogenesis (Table 1). The following integration of the data related to basal expression of miR-21, miR-17, and miR-155 showed no direct concordance with cellular responsiveness to µ-AMOs as well (Figure 2). However, we revealed a subtle trend showing that extremely high or low miRNA levels detected in lymphoid-derived tumour cells might be associated with low or no sensitivity to µ-AMO treatment (Figure 2). In turn, the efficacy of target miRNA inhibition might be the key factor, delineating the responsiveness of cell lines to µ-AMOs, targeted to miR-21, miR-17, and miR-155. Thus, the strongest suppression of miR-21, miR-17, and miR-155 was detected in the four most sensitive cell lines (HuTu-80, A549, CT-26, and Caco-2), whereas moderately sensitive lines KB-3-1, KB-8-5, Neuro-2a, and non-responding K562 cells demonstrated weaker and more variable responses (Figure 4 and Figure 5). It should be noted that the observed pattern of miRNA inhibition was assessed at a single time point and therefore may not fully reflect potential differences in miRNA turnover dynamics among the investigated cell types. Moreover, it should be noted that the observed decrease in miRNA levels detected by stem-loop RT-qPCR may reflect miRNA degradation, either by RNase H since mesyl oligonucleotide heteroduplexes are RNase H substrates [60], or/and by TDMD-like degradation pathways [69]. Both mechanisms may contribute to the observed decrease in the RT-qPCR signal. Nevertheless, the obtained data indicate that application of paired µ-AMO combinations revealed cell-specific response patterns, ranging from maintenance or enhancement of the effect to its attenuation or complete loss, supporting the idea that distinct molecular mechanisms underlie the response of each cell line to µ-AMO therapy.

The present study has several limitations. These include the absence of a detailed comparison between the efficacy of individual µ-AMOs and their paired combinations across cell lines of different histogenetic origins, the assessment of therapeutic effects at a single time point rather than in a dynamic setting, and the lack of in-depth investigation into the molecular mechanisms underlying the observed differences in sensitivity to µ-AMOs. However, it should be emphasised that this study was primarily designed to identify tumour types exhibiting the highest responsiveness to µ-AMO-based treatment and thereby establish the therapeutic settings in which the developed oligonucleotide combinations may demonstrate the highest efficacy. Whereas optimisation of concentration-dependent and kinetic parameters for each oligonucleotide individually, development of optimal therapeutic regimens, evaluation of potential synergistic interactions between oligonucleotide combinations, and investigation of the molecular reprogramming induced by µ-AMOs in tumour cells of different histogenetic origins remain subjects of dedicated future studies and represent a logical continuation of this line of research.

Taken together, the present study represents the first large-scale screening of mesyl phosphoramidate oligonucleotides targeting miR-21, miR-17, and miR-155, defining the most promising area of application for these compounds as tumours of epithelial origin, particularly intestinal and duodenal adenocarcinomas. These findings provide a foundation for further preclinical studies in this group of neoplasms, which remain difficult to treat despite their high worldwide incidence and mortality.

## 4. Materials and Methods

### 4.1. Oligonucleotide Synthesis

Mesyl phosphoramidate oligonucleotides (µ-AMOs) were synthesised on an automated DNA/RNA synthesiser ASM-800 (Biosset, Novosibirsk, Russia) on a scale of 0.2–0.4 μmol using conventionally protected (A^Bz^, C^Bz^, G^iBu^ and T) 5′-dimethoxytrityl (DMTr) 2′-deoxyribonucleotide β-cyanoethyl-N,N-diisopropyl 3′-phosphoramidites and 1000 Å Controlled Pore Glass (CPG) supports loaded with the respective protected deoxyribonucleosides (Sigma-Aldrich, St. Louis, MO, USA) according to the protocols described earlier [60]. All oligonucleotides used in the study are listed in Table 2. The sequences of µ-ASOs and qPCR primers were designed based on human miRNA sequences. The mature sequences of hsa-miR-21 and hsa-miR-17 are identical to their murine counterparts. For miR-155, a single nucleotide difference at position 12 between hsa-miR-155-5p and mmu-miR-155-5p was considered non-critical for µ-ASO binding efficacy and qPCR detection.

### 4.2. Cell Lines

The following cell lines were used in the study: (murine) melanoma B16, colon carcinoma CT-26 and lymphosarcoma RLS_40_, (human) epidermoid squamous carcinoma (epidermis) A431, lung adenocarcinoma A549, colorectal adenocarcinoma Caco-2, cervix epidermoid carcinoma Caski, cervical adenocarcinoma HeLa, foreskin fibroblasts hFF3, hepatocellular carcinoma HepG2, duodenal adenocarcinoma HuTu-80, T-cell acute lymphoblastic leukaemia Jurkat, chronic myeloid leukaemia K562, epidermoid carcinoma KB-3-1 and KB-8-5, neuroblastoma KELLY, breast adenocarcinoma MCF-7, neuroblastoma Neuro-2a, Burkitt lymphoma Raji, cervical squamous cell carcinoma SiHa, glioma U118, glioblastoma U87, and histiocytic lymphoma U937.

B16 cells were obtained from the Cell Culture Bank of the Blokhin National Medical Oncology Research Centre (Moscow, Russia). A549, MCF-7, HuTu-80, CT-26, Caco-2, KB-3-1, HeLa, Neuro-2a, HepG2, and K562 cells were purchased from the bank of cell cultures of the Institute of Cytology, Russian Academy of Sciences (St. Petersburg, Russia). KELLY, U937, and Jurkat cells were purchased from the European Collection of Authenticated Cell Cultures (ECACC), UK Health Security Agency (London, UK). RLS_40_ cells were obtained from the Institute of Chemical Biology and Fundamental Medicine, SB RAS (Novosibirsk, Russian Federation). hFF3 cells were kindly provided by Dr. O. A. Koval, Institute of Chemical Biology and Fundamental Medicine, SB RAS (Novosibirsk, Russia). A431 and U118 cells were kindly provided by Dr. E. P. Goncharova, Institute of Chemical Biology and Fundamental Medicine, SB RAS (Novosibirsk, Russia). U87 and Raji cells were kindly provided by Dr. A.P. Stepanov, Shemyakin–Ovchinnikov Institute of Bioorganic Chemistry RAS (Moscow, Russian Federation).

Cells were cultivated at 37 °C in a humidified incubator with 5% CO_2_ (standard conditions) in standard culture medium: Dulbecco’s modified Eagle’s medium (DMEM) (Sigma-Aldrich, St. Louis, MO, USA) (all cell lines except hFF3, Jurkat, K562, Raji, RLS_40_, and U937); Roswell Park Memorial Institute medium (RPMI) (Thermo Fisher Scientific, Waltham, MA, USA) (Jurkat, K562, Raji, and U937); or Iscove’s Modified Dulbecco’s Medium (IMDM) (Sigma-Aldrich, St. Louis, MO, USA) (hFF3 and RLS_40_) supplemented with 10% fetal bovine serum (FBS) (Thermo Fisher Scientific, Waltham, MA, USA) and 1% antibiotic-antimycotic solution (10,000 mg/mL streptomycin, 10,000 IU/mL penicillin, and 25 μg/mL amphotericin) (Biochemist, St. Petersburg, Russia).

### 4.3. Transfection of Tumour Cells with µ-AMOs

Cells were seeded into 96-well or 6-well plates at the density shown in Table 3, 24 h prior to transfection. To carry out transfection, the cationic liposomes 2X3-DOPE and µ-AMOs were mixed to form lipoplexes in serum-free Opti-MEM (Thermo Fisher Scientific, Waltham, MA, USA). Lipoplexes were prepared at an N/P ratio of 4/1, where N/P is the ratio between the nitrogen atoms of the polycationic lipid in liposomes and the phosphorus atoms in oligonucleotides. The resulting mixtures of 2X3-DOPE and µ-AMOs were incubated for 20 min at room temperature and added to the cells.

For cell growth analysis and migration studies, cells were transfected with pairwise combinations of µ-AMOs (µ-21 + µ-17/µ-21 + µ-155/µ-17 + µ-155) taken at a concentration of 60 nM of each µ-AMO (120 nM in total) or control µ-Scr (120 nM). To assess delivery efficiency, tumour cells were transfected with FAM-21 (Table 2) at a concentration of 150 nM. For evaluation of miRNA downregulation efficiency (PCR), cells were treated with either single µ-AMOs or µ-AMO pairs at a total concentration of 120 nM.

During the transfection, the cells were incubated at 37 °C in a humidified atmosphere with 5% CO_2_ for 4 h in Opti-Mem. The medium was then replaced with fresh standard culture medium, containing 10% FBS and 1% antibiotic antimycotic solution, and the cells were further cultivated for 0–72 h.

### 4.4. MTT/WST1 Test

The cell growth analysis following treatment with µ-AMOs was determined by the MTT test for monolayer cell lines including B16, CT-26, A431, A549, Caco-2, Caski, HeLa, hFF3, HepG2, HuTu-80, KB-3-1, KB-8-5, KELLY, MCF-7, Neuro-2a, SiHa, U118, and U87 and via the WST1 test for suspension cells such as RLS_40_, Jurkat, K562, Raji, and U937. Cells were transfected with µ-AMO pairs as described above. MTT solution (5 mg/mL) (Sigma-Aldrich, St. Louis, MO, USA) or WST1 solution (Abcam, Boston, MA, USA) at a dilution of 1:10 (vol.) was added to each well 72 h post-transfection, and cells were further incubated for 3 h under standard conditions. For the MTT test, crystals of formazan were dissolved in DMSO. The absorbance was measured using the Multiskan RC reader (LabSystems, Atlanta, GA, USA) at 570/620 nm (MTT) and 450/620 nm (WST1).

### 4.5. Migration Studies

Migration activity of B16, HuTu-80, KB-8-5, KB-3-1, Caco-2, and A549 cells was analysed by the scratch assay. A day before transfection, cells were seeded at the corresponding density per well (Table 3) in antibiotic-free DMEM into a 6-well plate. When cell confluency reached 80%, cells were incubated with mytomicin C (Sigma-Aldrich, St. Louis, MO, USA) (15 µg/mL) in antibiotic-free DMEM for 1 h in order to block the proliferation activity of tumour cells. At 1 h post-incubation, the medium was changed to serum-free and antibiotic-free DMEM and transfected with µ-ASO pairs at a total concentration of 120 nM (60 nM of each ASO) as described in Section 4.3. At 4 h post-transfection, the medium was changed to complete DMEM and incubated for 24 h. For all cells except KB-3-1 and KB-8-5, 24 h after transfection, three wound gaps in each well were scratched vertically with a micropipette tip. The floating cells were washed away twice with sterile phosphate-buffered saline (PBS) before adding complete DMEM. At 0, 24 h, or 48 h after scratching, the cells were photographed using a phase-contrast microscope (Zeiss Primo Vert, Carl Zeiss, Jena, Germany) and analysed with ImageJ software v. 1.54j (National Institutes of Health, Bethesda, MD, USA). Along the length of each scratch, at least five photographs were taken, and the width of each scratch was calculated as a mean value ± standard error of at least five measurements obtained for one scratch. Further, for each well, the average scratch width for the given time point was calculated as the mean value ± the standard error of three independent scratches inflicted in each well. The migration area was estimated as the ratio of the area filled with cells after 24 or 48 h to the initial scratch area. The migration rate of the cells was estimated as the degree of wound healing and calculated according to the formula: υ = (1 − Χ) × 100%, where Χ is the ratio of the scratch width at 24 or 48 h to the scratch width at 0 h.

Migration capacity of KB-8-5 and KB-3-1 cells was assessed using the real-time monitoring RTCA xCelligence system (ACEA Biosciences, Santa Clara, CA, USA). Tumour cells were transfected with µ-AMO pairs as described above and 24 h post-transfection detached using TrypLE (Thermo Fisher Scientific, Waltham, MA, USA), and seeded into the upper chamber of the CIM plate in serum-free DMEM at a density of 50,000 cells per well. Whereas the lower chamber was filled with DMEM, supplemented with 10% FBS, acting as a chemoattractant. Migration of tumour cells from the upper to the lower chamber was monitored during 72 h and was estimated as the percentage of tumour cells migrated relative to control untreated cells.

### 4.6. Transfection Efficiency Analysis

ASO delivery efficiency using 2X3-DOPE was measured using flow cytometry 4 h after transfection with FAM-21 (Table 2) (see above). Cells were harvested, washed with saline solution, resuspended, and fixed in 4% formaldehyde in PBS. Cells were analysed using the Novocyte 3000 (ACEA Biosciences, San Diego, CA, USA) flow cytometer (the excitation wavelength was 496 nm, and the emission wavelength was 519 ± 30 nm). Ten thousand events were collected for each sample. Two parameters reflecting the delivery efficiency were analysed: (1) the percentage of cells with green fluorescence exceeding the maximum level of the auto-fluorescence of untreated cells, and (2) the mean fluorescence intensity of cells measured in relative fluorescent units (‘RFU’).

### 4.7. qPCR

To assess the basal level of miRNA expression in intact cells and analyse the efficiency of miRNA downregulation 48 h post-transfection with µ-AMOs, total RNA was isolated using RIzol Reagent (DIAM, Moscow, Russia) according to the manufacturer’s protocol. The level of miRNAs was measured using stem-loop qPCR technology [70,71]. The RT and PCR primers used in the study are listed in Table 2. cDNA was synthesised using 3 μg of total cellular RNA in 20 μL reaction mixtures containing: reverse transcription buffer (1×) (Biolabmix, Novosibirsk, Russia), 50 nM reverse transcription-specific primer (Table 2), and 100 U of M-MuLV-RH reverse transcriptase (Biolabmix, Russia). Reverse transcription was performed as previously described [72,73]. PCR amplification was carried out using BioMaster HS-qPCR SYBR Blue mix (Biolabmix, Russia) according to the manufacturer’s protocol. The obtained qPCR data were analysed by the standard Bio-Rad iQ5 v.2.0 software. The ΔΔCt method was used to determine the relative miRNA levels, with U6 used for normalisation.

### 4.8. Statistics

The data were analysed using one-way ANOVA with post hoc Tukey’s test, using STATISTICA v. 6.0. Statistical significance was considered for *p* < 0.05. Prior to analysis, the data were tested for normality (Shapiro–Wilk test) and equality of variances (Brown–Forsythe test).

## Figures and Tables

**Table 1:** The efficiency of accumulation of FAM-labelled miR-21 specific oligonucleotide in different cells mediated by cationic liposomes 2X3-DOPE. Data are presented as mean ± SEM from at least three biological experiments.

Cell Line	% of Transfected Cells in Population	Mean Fluorescence Intensities, RFU × 10^5^
B16	96.5 ± 0.7	4.3 ± 0.3
HuTu-80	81.7 ± 2.5	3.3 ± 0.9
A549	82.7 ± 2.7	0.6 ± 0.05
CT-26	97.2 ± 1.3	3.4 ± 0.9
Caco-2	94.0 ± 1.3	3.8 ± 0.5
HeLa	94.1 ± 0.8	6.1 ± 1.8
Neuro-2a	87.5 ± 4.2	6.2 ± 2.7
KB-8-5	90.6 ± 4.7	1.2 ± 0.2
K562	86.7 ± 3.6	3.2 ± 1.1
Jurkat	87.5 ± 1.9	14.8 ± 0.2
Raji	85.7 ± 0.5	14.0 ± 0.5
hFF3	85.5 ± 0.3	15.5 ± 0.2

**Table 2:** Oligonucleotides and primers used in the study.

**ASO**	**Sequence 5′-3′**
μ-17	C^μ^T^μ^A^μ^C^μ^C^μ^T^μ^G^μ^C^μ^A^μ^C^μ^T^μ^G^μ^T^μ^A^μ^A^μ^G^μ^C^μ^A^μ^C^μ^T^μ^T^μ^T^μ^G
μ-21	T^μ^C^μ^A^μ^A^μ^C^μ^A^μ^T^μ^C^μ^A^μ^G^μ^T^μ^C^μ^T^μ^G^μ^A^μ^T^μ^A^μ^A^μ^G^μ^C^μ^T^μ^A
μ-155	A^μ^A^μ^C^μ^C^μ^C^μ^C^μ^T^μ^A^μ^T^μ^C^μ^A^μ^C^μ^G^μ^A^μ^T^μ^T^μ^A^μ^G^μ^C^μ^A^μ^T^μ^T^μ^A^μ^A
μ-Scr	C^μ^A^μ^A^μ^G^μ^T^μ^C^μ^T^μ^C^μ^G^μ^T^μ^A^μ^T^μ^G^μ^T^μ^A^μ^G^μ^T^μ^G^μ^G^μ^T^μ^T
FAM-21	FAM-TCAACATCAGTCTGATAAGCTA
**Primer**	**Sequence 5′-3′**
**Reverse transcription primers**	
RT-mir-17	GTCGTATCCAGTGCAGGGTCCGAGGTATTCGCACTGGATACGACCTACCTGCAC
RT-mir-155	GTCGTATCCAGTGCAGGGTCCGAGGTATTCGCACTGGATACGACGACACCCCTATCA
RT-mir-21	GTCGTATCCAGTGCAGGGTCCGAGGTATTCGCACTGGATACGACTCAACATCAG
RT-U6	GTCGTATCCAGTGCAGGGTCCGAGGTATTCGCACTGGATACGACAAAAATATGGAACG
**PCR primers**	
mir-17-F	AGACAAAGTGCTTACAGTGC
mir-155-F	ACTTAATGCTAATTGTGATAGG
mir-21-F	AGACTAGCTTATCAGACTGA
U6-F	CTCGCTTCGGCAGCACA
Universal reverse	GTGCAGGGTCCGAGGT

**Table 3:** Cell seeding conditions used in biological activity assays.

Cell Growth (MTT/WST1 Test)	
Cell Line	Cell Density per Well
B16	6 × 10^3^
CT-26	7 × 10^3^
RLS_40_	7 × 10^3^
A431	5 × 10^3^
A549	4 × 10^3^
Caco-2	7–8 × 10^3^
Caski	9 × 10^3^
HeLa	6 × 10^3^
hFF3	7 × 10^3^
HepG2	7 × 10^3^
HuTu-80	6 × 10^3^
Jurkat	8 × 10^3^
K562	5 × 10^3^
KB-3-1	3 × 10^3^
KB-8-5	3 × 10^3^
KELLY	7 × 10^3^
MCF-7	3 × 10^3^
Neuro-2a	6 × 10^3^
Raji	7 × 10^3^
SiHa	9 × 10^3^
U118	4 × 10^3^
U87	4 × 10^3^
U937	7 × 10^3^
**Migration (scratch assay)**	
**Cell line**	**Cell density per well**
B16	0.7 × 10^6^
CT-26	1.4 × 10^6^
Caco-2	1.0 × 10^6^
A549	0.7 × 10^6^
HuTu-80	1.0 × 10^6^
KB-8-5	0.4 × 10^6^
KB-3-1	0.4 × 10^6^

## Data Availability

The data generated or analysed during this study are included in this article or in the Supplementary Materials accompanying this paper. Further inquiries can be directed to the corresponding author.

## Supplementary Materials

The following supporting information can be downloaded at: https://www.mdpi.com/article/10.3390/ijms27125446/s1.

## Abbreviations

The following abbreviations are used in this manuscript:

AIF	Apoptosis-Inducing Factor
Akt	Protein Kinase B
anti-miRs	Anti-microRNAs
ASO	Antisense Oligonucleotide
BRG1	Brahma-Related Gene 1
CC BY	Creative Commons Attribution
CCNB1	Cyclin B1
CCND1	Cyclin D1
CCNG2	Cyclin G2
CDK1/2	Cyclin-Dependent Kinase 1/2
CDKN1B	Cyclin-Dependent Kinase Inhibitor 1B
CLL	Chronic Lymphocytic Leukaemia
CO_2_	Carbon Dioxide
CPG	Controlled Pore Glass
DEPTOR	DEP domain containing mTOR-interacting protein
DMEM	Dulbecco’s modified Eagle’s medium
DMSO	Dimethyl sulfoxide
DMTr	5′-dimethoxytrityl
E2F1	E2F Transcription Factor 1
ECACC	European Collection of Authenticated Cell Cultures
FBS	Fetal bovine serum
IMDM	Iscove’s Modified Dulbecco’s Medium
Jade-1	Jointly Acting on Dbl-L and E3b protein 1
LNA	Locked Nucleic Acid
MAP3K10	Mitogen-Activated Protein Kinase Kinase Kinase 10
miRNAs (miR)	Microribonucleic acids
mTOR	Mechanistic Target of Rapamycin
MTT	Methyltetrazolium test
µ	Mesyl
µ-AMOs	N-methanesulfonyl phosphoramidate (mesyl) anti-microRNA oligonucleotides
NF-κB	Nuclear Factor kappa B
p53	Tumour protein p53
PBS	Phosphate buffered saline
PDCD4	Programmed Cell Death protein 4
PIK3R1	Phosphoinositide-3-Kinase Regulatory Subunit 1
PS	phosphorothioate
PTEN	Phosphatase and Tensin Homolog
qPCR	Quantitative Polymerase Chain Reaction
RFU	Relative fluorescent units
RNase H	Ribonuclease H
RTCA	Real-Time Cell Analyzer
RT-PCR	Reverse Transcription Polymerase Chain Reaction
TGF-β	Transforming Growth Factor Beta
TGFBR2	Transforming Growth Factor Beta Receptor 2
TIMP2	Tissue Inhibitor of Metalloproteinase 2
TP53INP1	Tumour Protein p53 Inducible Nuclear Protein 1
TPM1	Tropomyosin 1
TRAF4	TNF Receptor Associated Factor 4
VEGF	Vascular Endothelial Growth Factor
WDR82	WD Repeat Domain 82
WEE1	WEE1 G2 checkpoint kinase
WST1	Water-soluble tetrazolium salt 1

## References

[1] Bray F., Laversanne M., Sung H., Ferlay J., Siegel R.L., Soerjomataram I., Jemal A. (2024). Global Cancer Statistics 2022: GLOBOCAN Estimates of Incidence and Mortality Worldwide for 36 Cancers in 185 Countries. CA. Cancer J. Clin..

[2] Reynolds I.S., Fichtner M., McNamara D.A., Kay E.W., Prehn J.H.M., Burke J.P. (2019). Mucin Glycoproteins Block Apoptosis; Promote Invasion, Proliferation, and Migration; and Cause Chemoresistance through Diverse Pathways in Epithelial Cancers. Cancer Metastasis Rev..

[3] Dotto G.P. (2014). Multifocal Epithelial Tumors and Field Cancerization: Stroma as a Primary Determinant. J. Clin. Investig..

[4] Nemkov T., D’Alessandro A., Reisz J.A. (2019). Metabolic Underpinnings of Leukemia Pathology and Treatment. Cancer Rep..

[5] Whiteley A.E., Price T.T., Cantelli G., Sipkins D.A. (2021). Leukaemia: A Model Metastatic Disease. Nat. Rev. Cancer.

[6] Bale T.A., Rosenblum M.K. (2022). The 2021 WHO Classification of Tumors of the Central Nervous System: An Update on Pediatric Low-Grade Gliomas and Glioneuronal Tumors. Brain Pathol..

[7] Tsubota S., Kadomatsu K. (2018). Origin and Initiation Mechanisms of Neuroblastoma. Cell Tissue Res..

[8] Shang R., Lee S., Senavirathne G., Lai E.C. (2023). MicroRNAs in Action: Biogenesis, Function and Regulation. Nat. Rev. Genet..

[9] Catalanotto C., Cogoni C., Zardo G. (2016). MicroRNA in Control of Gene Expression: An Overview of Nuclear Functions. Int. J. Mol. Sci..

[10] Nalavade R., Singh M. (2023). Intracellular Compartmentalization: A Key Determinant of MicroRNA Functions. MicroRNA.

[11] Bautista-Sánchez D., Arriaga-Canon C., Pedroza-Torres A., De La Rosa-Velázquez I.A., González-Barrios R., Contreras-Espinosa L., Montiel-Manríquez R., Castro-Hernández C., Fragoso-Ontiveros V., Álvarez-Gómez R.M. (2020). The Promising Role of MiR-21 as a Cancer Biomarker and Its Importance in RNA-Based Therapeutics. Mol. Ther. Nucleic Acids.

[12] Zhao W., Gupta A., Krawczyk J., Gupta S. (2022). The MiR-17-92 Cluster: Yin and Yang in Human Cancers. Cancer Treat. Res. Commun..

[13] Wu Y., Hong Q., Lu F., Zhang Z., Li J., Nie Z., He B. (2023). The Diagnostic and Prognostic Value of MiR-155 in Cancers: An Updated Meta-Analysis. Mol. Diagn. Ther..

[14] Seyhan A.A. (2024). Trials and Tribulations of MicroRNA Therapeutics. Int. J. Mol. Sci..

[15] Gao Z., Liu H., Shi Y., Yin L., Zhu Y., Liu R. (2019). Identification of Cancer Stem Cell Molecular Markers and Effects of Hsa-Mir-21-3p on Stemness in Esophageal Squamous Cell Carcinoma. Cancers.

[16] Irimie-Aghiorghiesei A.I., Pop-Bica C., Pintea S., Braicu C., Cojocneanu R., Zimța A.A., Gulei D., Slabý O., Berindan-Neagoe I. (2019). Prognostic Value of Mir-21: An Updated Meta-Analysis in Head and Neck Squamous Cell Carcinoma (Hnscc). J. Clin. Med..

[17] Shen Z., Xu X., Lv L., Dai H., Chen J., Chen B. (2020). MiR-21 Overexpression Promotes Esophageal Squamous Cell Carcinoma Invasion and Migration by Repressing Tropomyosin 1. Gastroenterol. Res. Pract..

[18] Li X., Chen D., Li M., Gao X., Shi G., Zhao H. (2018). The CADM2/Akt Pathway Is Involved in the Inhibitory Effect of MiR-21-5p Downregulation on Proliferation and Apoptosis in Esophageal Squamous Cell Carcinoma Cells. Chem. Biol. Interact..

[19] Ishinaga H., Okugawa Y., Hou B., He F., Yin C., Murata M., Toiyama Y., Takeuchi K. (2023). The Role of MiR-21 as a Predictive Biomarker and a Potential Target to Improve the Effects of Chemoradiotherapy against Head and Neck Squamous Cell Carcinoma. J. Radiat. Res..

[20] Zheng Y., Xie J., Jiang F., Li Y., Chang G., Ma H. (2018). Inhibition of MiR-21 Promotes Cell Apoptosis in Oral Squamous Cell Carcinoma by Upregulating PTEN. Oncol. Rep..

[21] Musilova K., Mraz M. (2015). MicroRNAs in B-Cell Lymphomas: How a Complex Biology Gets More Complex. Leukemia.

[22] Chen C., Zhu F., Liu F., Yao Y., Ma Z., Luo S. (2023). Human Bone Marrow Mesenchymal Stem Cells-Derived Exosomal MiRNA-21-5p Inhibits Lidocaine-Induced Apoptosis in SH-SY5Y Neuroblastoma Cells. Iran. J. Public Health.

[23] Challagundla K.B., Wise P.M., Neviani P., Chava H., Murtadha M., Xu T., Kennedy R., Ivan C., Zhang X., Vannini I. (2015). Exosome-Mediated Transfer of MicroRNAs Within the Tumor Microenvironment and Neuroblastoma Resistance to Chemotherapy. J. Natl. Cancer Inst..

[24] Wang Z., Yao W., Li K., Zheng N., Zheng C., Zhao X., Zheng S. (2017). Reduction of MiR-21 Induces SK-N-SH Cell Apoptosis and Inhibits Proliferation via PTEN/PDCD4. Oncol. Lett..

[25] Wang J.X., Jia X.J., Liu Y., Dong J.H., Ren X.M., Xu O., Liu S.H., Shan C.G. (2020). Silencing of MiR-17-5p Suppresses Cell Proliferation and Promotes Cell Apoptosis by Directly Targeting PIK3R1 in Laryngeal Squamous Cell Carcinoma. Cancer Cell Int..

[26] Wang X., Han J., Liu Y., Hu J., Li M., Chen X., Xu L. (2021). MiR-17-5p and MiR-4443 Promote Esophageal Squamous Cell Carcinoma Development by Targeting TIMP2. Front. Oncol..

[27] Sun Y., Nie W., Qiu B., Guo X., Zhang J., Wei J. (2021). Inhibition of MicroRNA-17 Enhances Cisplatin-Induced Apoptosis of Human Tongue Squamous Carcinoma Cell. J. Bioenerg. Biomembr..

[28] Jing C., Ma G., Li X., Wu X., Huang F., Liu K., Liu Z. (2016). MicroRNA-17/20a Impedes Migration and Invasion via TGF-β/ITGB6 Pathway in Esophageal Squamous Cell Carcinoma. Am. J. Cancer Res..

[29] Huang Q., Shen Y.J., Hsueh C.Y., Guo Y., Zhang Y.F., Li J.Y., Zhou L. (2021). MiR-17-5p Drives G2/M-Phase Accumulation by Directly Targeting CCNG2 and Is Related to Recurrence of Head and Neck Squamous Cell Carcinoma. BMC Cancer.

[30] Chen W., Hao X., Yang B., Zhang Y., Sun L., Hua Y., Yang L., Yu J., Zhao J., Hou L. (2021). MYCN-Amplified Neuroblastoma Cell-Derived Exosomal MiR-17-5p Promotes Proliferation and Migration of Non-MYCN Amplified Cells. Mol. Med. Rep..

[31] Gruszka R., Zakrzewski K., Liberski P.P., Zakrzewska M. (2020). MicroRNA Interaction with MAPK and AKT Pathways in Paediatric Brain Tumours-Preliminary Results and Review of the Literature. Folia Neuropathol..

[32] Gruszka R., Zakrzewski J., Nowosławska E., Grajkowska W., Zakrzewska M. (2024). Identification and Validation of MiRNA-Target Genes Network in Pediatric Brain Tumors. Sci. Rep..

[33] Gruszka R., Zakrzewski K., Liberski P.P., Zakrzewska M. (2021). Mrna and Mirna Expression Analyses of the Myc/E2f/Mir-17-92 Network in the Most Common Pediatric Brain Tumors. Int. J. Mol. Sci..

[34] Murphy B.L., Obad S., Bihannic L., Ayrault O., Zindy F., Kauppinen S., Roussel M.F. (2013). Silencing of the MiR-17~92 Cluster Family Inhibits Medulloblastoma Progression. Cancer Res..

[35] Kalantzakos T., Hooper K., Das S., Sullivan T., Canes D., Moinzadeh A., Rieger-Christ K. (2023). MicroRNA-155-5p Targets JADE-1, Promoting Proliferation, Migration, and Invasion in Clear Cell Renal Cell Carcinoma Cells. Int. J. Mol. Sci..

[36] Fu X., Wen H., Jing L., Yang Y., Wang W., Liang X., Nan K., Yao Y., Tian T. (2017). MicroRNA-155-5p Promotes Hepatocellular Carcinoma Progression by Suppressing PTEN through the PI3K/Akt Pathway. Cancer Sci..

[37] Zhou R., Chen Z., Cai Y., Zhang H., Mao S., Zhuang Y., Zheng J. (2024). The Simultaneous MiR-155-5p Overexpression and MiR-223-3p Inhibition Can Activate PEMT in Oral Squamous Cell Carcinoma. J. Appl. Oral Sci..

[38] Kong X., Liu F., Gao J. (2016). MiR-155 Promotes Epithelial-Mesenchymal Transition in Hepatocellular Carcinoma Cells through the Activation of PI3K/SGK3/β-Catenin Signaling Pathways. Oncotarget.

[39] Liu B., Hu J., Zhao H., Zhao L., Pan S. (2022). MicroRNA-155-5p Contributes to 5-Fluorouracil Resistance Through Down-Regulating TP53INP1 in Oral Squamous Cell Carcinoma. Front. Oncol..

[40] Luo W., Zhang H., Liang X., Xia R., Deng H., Yi Q., Lv L., Qian L. (2020). DNA Methylation–Regulated MiR–155–5p Depresses Sensitivity of Esophageal Carcinoma Cells to Radiation and Multiple Chemotherapeutic Drugs via Suppression of MAP3K10. Oncol. Rep..

[41] Fu S., Chen H.H., Cheng P., Zhang C.B., Wu Y. (2017). MiR-155 Regulates Oral Squamous Cell Carcinoma Tca8113 Cell Proliferation, Cycle, and Apoptosis via Regulating P27Kip1. Eur. Rev. Med. Pharmacol. Sci..

[42] Belnap C., Divis T., Kingsley K., Howard K.M. (2024). Differential Expression of MicroRNA MiR-145 and MiR-155 Downstream Targets in Oral Cancers Exhibiting Limited Chemotherapy Resistance. Int. J. Mol. Sci..

[43] Lei Q.Q., Huang Y., Li B., Han L., Lv C. (2020). MiR-155-5p Promotes Metastasis and Epithelial–Mesenchymal Transition of Renal Cell Carcinoma by Targeting Apoptosis-Inducing Factor. Int. J. Biol. Markers.

[44] Liu F., Mao Q., Zhu S., Qiu J. (2021). MicroRNA-155-5p Promotes Cell Proliferation and Invasion in Lung Squamous Cell Carcinoma through Negative Regulation of Fibroblast Growth Factor 9 Expression. J. Thorac. Dis..

[45] Wu H., Wu H., Sun P., Zhu D., Ma M., Fan W. (2021). MiR-155-5p Promotes Cell Proliferation and Migration of Clear Cell Renal Cell Carcinoma by Targeting PEG3. Urol. Int..

[46] Zhang X., Li M., Zuo K., Li D., Ye M., Ding L., Cai H., Fu D., Fan Y., Lv Z. (2013). Upregulated MiR-155 in Papillary Thyroid Carcinoma Promotes Tumor Growth by Targeting APC and Activating Wnt/β-Catenin Signaling. J. Clin. Endocrinol. Metab..

[47] Kirave P., Gondaliya P., Kulkarni B., Rawal R., Garg R., Jain A., Kalia K. (2020). Exosome Mediated MiR-155 Delivery Confers Cisplatin Chemoresistance in Oral Cancer Cells via Epithelial-Mesenchymal Transition. Oncotarget.

[48] Mikamori M., Yamada D., Eguchi H., Hasegawa S., Kishimoto T., Tomimaru Y., Asaoka T., Noda T., Wada H., Kawamoto K. (2017). MicroRNA-155 Controls Exosome Synthesis and Promotes Gemcitabine Resistance in Pancreatic Ductal Adenocarcinoma. Sci. Rep..

[49] Jabłońska E., Białopiotrowicz E., Szydłowski M., Prochorec-Sobieszek M., Juszczyński P., Szumera-Ciećkiewicz A. (2020). DEPTOR Is a MicroRNA-155 Target Regulating Migration and Cytokine Production in Diffuse Large B-Cell Lymphoma Cells. Exp. Hematol..

[50] Huang X., Shen Y., Liu M., Bi C., Jiang C., Iqbal J., McKeithan T.W., Chan W.C., Ding S.J., Fu K. (2012). Quantitative Proteomics Reveals That MiR-155 Regulates the PI3K-AKT Pathway in Diffuse Large B-Cell Lymphoma. Am. J. Pathol..

[51] Pan Y., Cengiz R., Kluiver J., Diepstra A., Van den Berg A. (2024). Pinpointing Functionally Relevant MiRNAs in Classical Hodgkin Lymphoma Pathogenesis. Cancers.

[52] Chang Y., Cui M., Fu X., Zhang L., Li X., Li L., Wu J., Sun Z., Zhang X., Li Z. (2019). MiRNA-155 Regulates Lymphangiogenesis in Natural Killer/T-Cell Lymphoma by Targeting BRG1. Cancer Biol. Ther..

[53] Park B., Choi M.E., Ryu K.J., Park C., Choi M., Yoon S.E., Kim W.S., Kim H.H., Hong J.Y., Kim S.J. (2024). Exosomal MiR-155-5p Drives Ibrutinib Resistance in B-Cell Lymphoma. Exp. Cell Res..

[54] Song L., Luan B., Xu Q., Shi R., Wang X. (2022). MicroRNA-155-3p Delivered by M2 Macrophages-Derived Exosomes Enhances the Progression of Medulloblastoma through Regulation of WDR82. J. Transl. Med..

[55] Chiglintseva D.A., Patutina O.A., Zenkova M.A. (2025). Endogenous Ribonucleases: Therapeutic Targeting of the Transcriptome Through Oligonucleotide-Triggered RNA Inactivation. Biomolecules.

[56] Patutina O., Miroshnichenko S., Chiglintseva D., Zenkova M. (2025). Opening New Frontiers with Catalytic Nucleic Acids in MiRNA Inhibition. Front. Pharmacol..

[57] Seto A.G., Beatty X., Lynch J.M., Hermreck M., Tetzlaff M., Duvic M., Jackson A.L. (2018). Cobomarsen, an Oligonucleotide Inhibitor of MiR-155, Co-Ordinately Regulates Multiple Survival Pathways to Reduce Cellular Proliferation and Survival in Cutaneous T-Cell Lymphoma. Br. J. Haematol..

[58] Anastasiadou E., Seto A.G., Beatty X., Hermreck M., Gilles M.E., Stroopinsky D., Pinter-Brown L.C., Pestano L., Marchese C., Avigan D. (2021). Cobomarsen, an Oligonucleotide Inhibitor of MiR-155, Slows DLBCL Tumor Cell Growth In Vitro and In Vivo. Clin. Cancer Res..

[59] Tassone P., Di Martino M.T., Arbitrio M., Fiorillo L., Staropoli N., Ciliberto D., Cordua A., Scionti F., Bertucci B., Salvino A. (2023). Safety and Activity of the First-in-Class Locked Nucleic Acid (LNA) MiR-221 Selective Inhibitor in Refractory Advanced Cancer Patients: A First-in-Human, Phase 1, Open-Label, Dose-Escalation Study. J. Hematol. Oncol..

[60] Miroshnichenko S.K., Patutina O.A., Burakova E.A., Chelobanov B.P., Fokina A.A., Vlassov V.V., Altman S., Zenkova M.A., Stetsenko D.A. (2019). Mesyl Phosphoramidate Antisense Oligonucleotides as an Alternative to Phosphorothioates with Improved Biochemical and Biological Properties. Proc. Natl. Acad. Sci. USA.

[61] Patutina O.A., Gaponova (Miroshnichenko) S.K., Sen’kova A.V., Savin I.A., Gladkikh D.V., Burakova E.A., Fokina A.A., Maslov M.A., Shmendel’ E.V., Wood M.J.A. (2020). Mesyl Phosphoramidate Backbone Modified Antisense Oligonucleotides Targeting MiR-21 with Enhanced in Vivo Therapeutic Potency. Proc. Natl. Acad. Sci. USA.

[62] Gaponova S., Patutina O., Sen’kova A., Burakova E., Savin I., Markov A., Shmendel E., Maslov M., Stetsenko D., Vlassov V. (2022). Single Shot vs. Cocktail: A Comparison of Mono- and Combinative Application of MiRNA-Targeted Mesyl Oligonucleotides for Efficient Antitumor Therapy. Cancers.

[63] Miroshnichenko S.K., Patutina O.A., Markov A.V., Kupryushkin M.S., Vlassov V.V., Zenkova M.A. (2025). Biological Performance and Molecular Mechanisms of Mesyl MicroRNA-Targeted Oligonucleotides in Colorectal Cancer Cells. Int. J. Mol. Sci..

[64] Patutina O., Sen’kova A., Miroshnichenko S., Awad M., Markov O., Gladkikh D., Savin I., Seroklinova E., Zhukov S., Kupryushkin M. (2026). Targeted Inhibition of Oncogenic MicroRNAs MiR-21, MiR-17, and MiR-155 Suppresses Tumor Growth and Modulates Immune Response in Colorectal Cancer. Pharmaceutics.

[65] Miroshnichenko S., Demirel R., Moralev A., Almieva O., Markov A., Burakova E., Stetsenko D., Maslov M., Vlassov V., Zenkova M. (2025). Reversing the Irreversible: MiRNA-Targeting Mesyl Phosphoramidate Oligonucleotides Restore Sensitivity to Cisplatin and Doxorubicin of KB-8-5 Epidermoid Carcinoma Cells. Biomedicines.

[66] Kabilova T.O., Shmendel E.V., Gladkikh D.V., Chernolovskaya E.L., Markov O.V., Morozova N.G., Maslov M.A., Zenkova M.A. (2018). Targeted Delivery of Nucleic Acids into Xenograft Tumors Mediated by Novel Folate-Equipped Liposomes. Eur. J. Pharm. Biopharm..

[67] Keller H., Yunxu C., Marit G., Pla M., Reiffers J., Thèze J., Froussard P. (1999). Transgene Expression, but Not Gene Delivery, Is Improved by Adhesion-Assisted Lipofection of Hematopoietic Cells. Gene Ther..

[68] Chasseval R.D., de Villartay J. (1992). pierre High Level Transient Gene Expression in Human Lymphoid Cells by SV40 Large T Antigen Boost. Nucleic Acids Res..

[69] Haas G., Cetin S., Messmer M., Chane-Woon-Ming B., Terenzi O., Chicher J., Kuhn L., Hammann P., Pfeffer S. (2016). Identification of Factors Involved in Target RNA-Directed MicroRNA Degradation. Nucleic Acids Res..

[70] Chen C., Ridzon D.A., Broomer A.J., Zhou Z., Lee D.H., Nguyen J.T., Barbisin M., Xu N.L., Mahuvakar V.R., Andersen M.R. (2005). Real-Time Quantification of MicroRNAs by Stem-Loop RT-PCR. Nucleic Acids Res..

[71] Varkonyi-Gasic E., Hellens R.P. (2011). Quantitative Stem-Loop RT-PCR for Detection of MicroRNAs. Methods Mol. Biol..

[72] Patutina O.A., Bichenkova E.V., Miroshnichenko S.K., Mironova N.L., Trivoluzzi L.T., Burusco K.K., Bryce R.A., Vlassov V.V., Zenkova M.A. (2017). MiRNases: Novel Peptide-Oligonucleotide Bioconjugates That Silence MiR-21 in Lymphosarcoma Cells. Biomaterials.

[73] Patutina O.A., Bazhenov M.A., Miroshnichenko S.K., Mironova N.L., Pyshnyi D.V., Vlassov V.V., Zenkova M.A. (2018). Peptide-Oligonucleotide Conjugates Exhibiting Pyrimidine-X Cleavage Specificity Efficiently Silence MiRNA Target Acting Synergistically with RNase H. Sci. Rep..

