# Supplementary material for: Panel-Wide Screening of Tumour Cells of Diverse Histogenesis for Responsiveness to Silencing of miR-21, miR-17, and miR-155 by Mesyl Phosphoramidate Antisense Oligonucleotides
Source: Int J Mol Sci. 2026 Jun 16;27(12):5446. doi: 10.3390/ijms27125446 (PMC13299803; doi:10.3390/ijms27125446)
Supplement: Supplementary file 1 [file ijms-27-05446-s001.zip › ijms-4356696-supplementary.pdf]

# Supplementary material

## Panel-wide screening of tumor cells of diverse histogenesis for responsiveness to silencing of miR-21, miR-17, and miR-155 by mesyl phosphoramidate antisense oligonucleotides

Svetlana Miroshnichenko[1], Olga Patutina[1], Olga Almieva[1], Ekaterina Burakova[2,3], Mikhail Maslov[4], Alesya Fokina[2,3], Dmitry Stetsenko[2,3] and Marina Zenkova[1*]

[1] Institute of Chemical Biology and Fundamental Medicine SB RAS, Novosibirsk 630090, Russia;
sveta-mira@yandex.ru (S.M.); patutina@1bio.ru (O.P.); yakovenko0lya@yandex.ru (O.A.)
[2] Department of Physics, Novosibirsk State University, Novosibirsk 630090, Russia;
ekaanabur@yandex.ru (E.B.); a.fokina@nsu.ru (A.F.); d.stetsenko@nsu.ru (D.S.)
[3] Institute of Cytology and Genetics SB RAS, Novosibirsk 630090, Russia
[4] Lomonosov Institute of Fine Chemical Technologies, MIREA—Russian Technological University,
Moscow 119571, Russia; mamaslov@mail.ru
* Correspondence: marzen@1bio.ru or marzen@niboch.nsc.ru; Tel.: +7-383-363-51-60

Table of consent

Table S1. Type and origin of cell lines investigated in the current study..……………………………………..2

Figure S1 Dose-dependent anti-proliferative effects of μ-21, μ-17, and μ-155 on B16, CT-26, Caco-2, and KB-8-5 tumor cells………………………………………………….……………………………………...3

Table S2. Complete dataset showing anti-proliferative effects of paired combinations of μ-AMOs, targeted to miR-21, miR-17, and miR-155 on tumor cells viability ...……………………………………………...4

Table S3. Effects of control μ-Scr oligonucleotide on tumor cells viability (% relative to Control)………………………………………………………………….............................…………….……...5

Table S4. Effects of mono and pairwise combinations of μ-AMOs on cell growth of tumour cells with high or moderate sensitivity to μ-AMOs (% relative to Control)………………………..............…………………...5

Figure S2. Inhibition of migrative activity of tumor cells by pairwise combinations of μ-AMOs, targeted to miR-21, miR-17 and miR-155……………………………………….............................…………….……...6

Figure S3. Basal expression of miR-21, miR-17 and miR-155 in all studied cell lines..............…………………...7

**Table S1.** Type and origin of cell lines investigated in the current study.

| Cell line | Type of neoplasia | Origin |
|---|---|---|
| **Epithelial origin** | | |
| A431 | Epidermoid (skin) carcinoma | *H. sapiens* |
| A549 | Lung adenocarcinoma | *H. sapiens* |
| Caco-2 | Colorectal adenocarcinoma | *H. sapiens* |
| Caski | Cervical carcinoma | *H. sapiens* |
| CT-26 | Intestinal adenocarcinoma | *M. musculus* |
| HeLa | Cervical carcinoma | *H. sapiens* |
| HepG2 | Hepatocellular carcinoma | *H. sapiens* |
| HuTu-80 | Duodenal adenocarcinoma | *H. sapiens* |
| KB-3-1 | Epidermoid (cervical) carcinoma | *H. sapiens* |
| KB-8-5 | Epidermoid (cervical) carcinoma* | *H. sapiens* |
| MCF-7 | Breast adenocarcinoma | *H. sapiens* |
| SiHa | Cervical carcinoma | *H. sapiens* |
| **Lymphoid origin** | | |
| Jurkat | T cell leukemia | *H. sapiens* |
| K562 | Chronic myeloid leukemia | *H. sapiens* |
| Raji | Burkitt lymphoma | *H. sapiens* |
| $RLS_{40}$ | Lymphosarcoma* | *M.musculus* |
| U937 | Histiocytic lymphoma | *H. sapiens* |
| **Melanocytic origin** | | |
| B16 | Melanoma | *M.musculus* |
| **Neuronal and glial origin** | | |
| KELLY | Neuroblastoma | *H. sapiens* |
| Neuro-2a | Neuroblastoma | *M.musculus* |
| U118 | Glioblastoma | *H. sapiens* |
| U87 | Glioblastoma | *H. sapiens* |
| **Connective tissue / mesenchymal origin** | | |
| hFF3 | Non-transformed fibroblasts | *H. sapiens* |

* - tumor cell line exhibiting stepwise-selected multidrug resistant phenotype

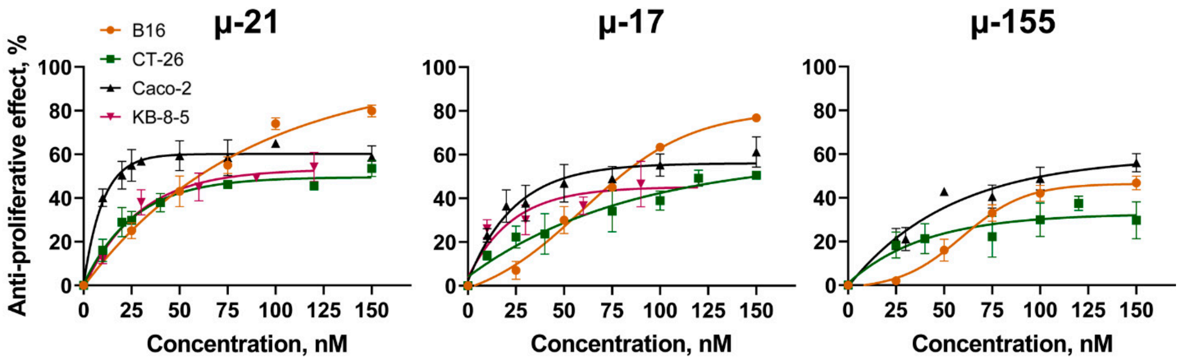


**Figure S1.** Dose-dependent anti-proliferative effects of μ-21, μ-17, and μ-155 on B16, CT-26, Caco-2, and KB-8-5 tumor cells. Results of MTT assay 72 h post-transfection with single μ-AMOs in 10-150 nM concentration. Data are presented as mean±SEM from at least three biological experiments and three technical replicates.

**Table S2.** Complete dataset showing anti-proliferative effects of paired combinations of μ-AMOs, targeted to miR-21, miR-17, and miR-155 on tumor cells growth.

| Tumor cell line | Effect on cell viability, % | | |
|---|---|---|---|
| | μ-21 + μ-17 | μ-21 + μ-155 | μ-17 + μ-155 |
| **B16** | 71.5 ± 5.2** # | 71.8 ± 6.4*** # | 70.8 ± 8.1** |
| **HuTu-80** | 74.4 ± 3.5*** | 69.1 ± 3.5*** | 69.7 ± 1.6*** |
| **A549** | 51.8 ± 3.2*** | 50.8 ± 3.2*** | 48.1 ± 1.5*** |
| **CT-26** | 43.8 ± 2.9*** ### | 44.5 ± 1.8*** ### | 42.4 ± 2.4*** ### |
| **Caco-2** | 46.4 ± 4.1*** # | 50.4 ± 0.6*** ## | 31.8 ± 6.0** # |
| **KB-3-1** | 45.6 ± 12.1 | 43.5 ± 4.1** | 34.8 ± 1.72** |
| **MCF-7** | 45.8 ± 3.0*** ## | 37.6 ± 1.9*** # | 36.2 ± 1.6*** # |
| **A431** | 59.0 ± 10.3* | 28.6 ± 1.1** | 35.5 ± 2.3** |
| **HeLa** | 40.7 ± 2.7*** | 43.2 ± 6.2** | 34.8 ± 3.4*** |
| **$RLS_{40}$** | 37.4 ± 3.3** | 44.2 ± 7.9* | 28.5 ± 6.3 |
| **U118** | 31.3 ± 5.0** # | 36.4 ± 4.4** ## | 58.1 ± 2.2*** ### |
| **Neuro-2a** | 23.8 ± 2.4*** | 37.0 ± 4.1*** ## | 45.5 ± 8.0*** ## |
| **KB-8-5** | 43.5 ± 4.7* | 38.1 ± 6.6* | 22.4 ± 17.2 |
| **HepG2** | 36.7 ± 3.1*** ## | 36.9 ± 3.4*** ## | 21.0 ± 4.7** |
| **U87** | 40.4 ± 1.4*** ## | 28.9 ± 3.9** | 12.6 ± 2.1**# |
| **Caski** | 18.3 ± 1.5* | 15.8 ± 2.2* | 25.6 ± 4.3* |
| **SiHa** | 27.5 ± 9.3 | 18.6 ± 5.1* | 10.5 ± 8.0 |
| **KELLY** | 15.0 ± 1.4*** ## | 16.3 ± 2.2** ## | 17.3 ± 2.1** ## |
| **U937** | 15.3 ± 3.4* | 24.6 ± 14.2 | 8.0 ± 1.9 |
| **Jurkat** | 4.9 ± 4.2 | 12.0 ± 7.9 | 0.0 ± 2.4 |
| **Raji** | 0.0 ± 3.6 | 6.3 ± 1.5 | 10.9 ± 6.9 |
| **K562** | 0.0 ± 2.3 | 0.0 ± 2.1 | 0.0 ± 1.2 |
| **hFF3** | 0.0 ± 0.7 | 0.0 ± 0.6 | 0.0 ± 1.4 |

*The effects are presented in % relative to untreated Control. Data are presented as mean±SEM from at least three biological experiments and three technical replicates.*

*, **, *** – *statistically significant differences from Control with p-value <0.05. <0.01 and <0.001. respectively;*

#, ##, ### – *statistically significant differences from μ-Scr with p-value <0.05. <0.01 and <0.001. respectively.*

**Table S3.** Effects of control μ-Scr oligonucleotide on tumor cells viability for cell lines of different sensitivity.

| Tumor cell line | Effect on cell viability, % | Sensitivity to μ-AMO pairs |
|---|---|---|
| **CT-26** | 18.7 ± 2.4 | Top 5 most sensitive |
| **Caco-2** | 16.2 ± 5.4 | Top 5 most sensitive |
| **U118** | 3.9 ± 5.3 | Moderate |
| **Neuro-2a** | 10.3 ± 7.6 | Moderate |
| **HepG2** | 19.1 ± 5.1 | Moderate |
| **U87** | 19.3 ± 0.5 | Moderate |
| **Caski** | 13.3 ± 5.9 | Low |
| **SiHa** | 12.0 ± 5.6 | Low |
| **KELLY** | 0.0 ± 3.2 | Low |
| **Jurkat** | 8.2 ± 5.9 | Low |
| **Raji** | 2.9 ± 0.95 | Low |
| **K562** | 1.9 ± 0.18 | No response |
| **hFF3** | 0.0 ± 1.5 | No response |

*The effects are presented in % relative to untreated Control. Data are presented as mean±SEM from at least three biological experiments and three technical replicates.*

**Table S4.** Effects of mono and pairwise combinations of μ-AMOs on cell growth of tumour cells with high or moderate sensitivity to μ-AMOs.

| Tumor cell line | Effect on cell viability, % | | | | | |
|---|---|---|---|---|---|---|
| | **μ-21** | **μ-17** | **μ-155** | **μ-21 + μ-17** | **μ-21 + μ-155** | **μ-17 + μ-155** |
| **B16** | 74.0±2.7 | 45.0±1.9 | 42.0±3.7 | 71.5 ± 5.2 | 71.8 ± 6.4 | 70.8 ± 8.1 |
| **A549** | 36.3±0.5 | 49.7±1.7 | 41.5±1.4 | 51.8 ± 3.2 | 50.8 ± 3.2 | 48.1 ± 1.5 |
| **CT-26** | 45.5±0.8 | 49.2±3.7 | 37.4±3.3 | 43.8 ± 2.9 | 44.5 ± 1.8 | 42.4 ± 2.4 |
| **Caco-2** | 65.0±1.2 | 55.2±5.1 | 48.8±5.1 | 46.4 ± 4.1 | 50.4 ± 0.6 | 31.8 ± 6.0 |
| **U118** | 14.1±4.6 | 10.1±1.4 | 0.8±4.0 | 31.3 ± 5.0 [#] | 36.4 ± 4.4 | 58.1 ± 2.2 |
| **KB-8-5** | 54.2±6.7 | 48.2±2.6 | 41.3±7.5 | 43.5 ± 4.7 | 38.1 ± 6.6 | 22.4 ± 17.2 |

*The effects are presented in % relative to untreated Control. Data are presented as mean±SEM from at least three biological experiments and three technical replicates. μ-AMOs – cells transfected with individual oligonucleotides (120 nM) or pairs (60 nM each AMO, total concentration 120 nM) in complex with 2X3-DOPE.*

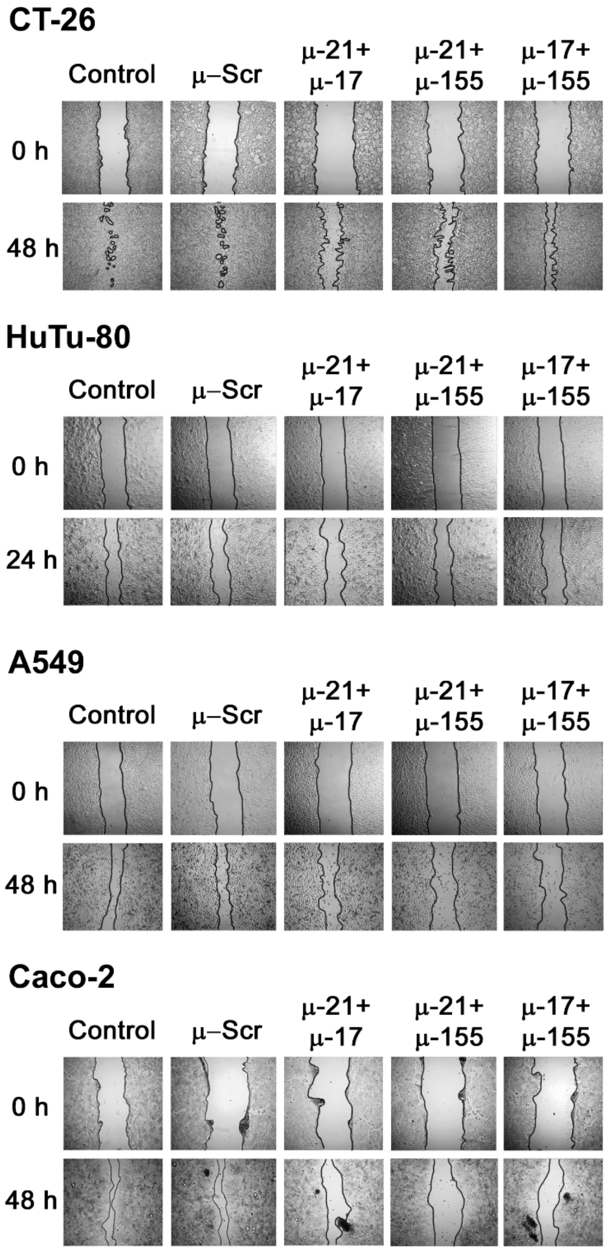


**Figure S2.** Inhibition of migratory activity of tumor cells by μ-AMOs pairs, targeted to miR-21, miR-17 and miR-155. Photographs of wounds in CT-26, HuTu-80, A549, and Caco-2 cell monolayers, at 0 and 24 h/ 48 h after transfection with μ-ASOs. 4-fold magnification. Black lines show wound borderlines. Control – intact tumor cells; μ-Scr, μ-21+μ-17, μ-21+μ-155 and μ-17+μ-155 – cells transfected with corresponding oligonucleotides in total 120 nM concentration in complex with 2X3-DOPE.

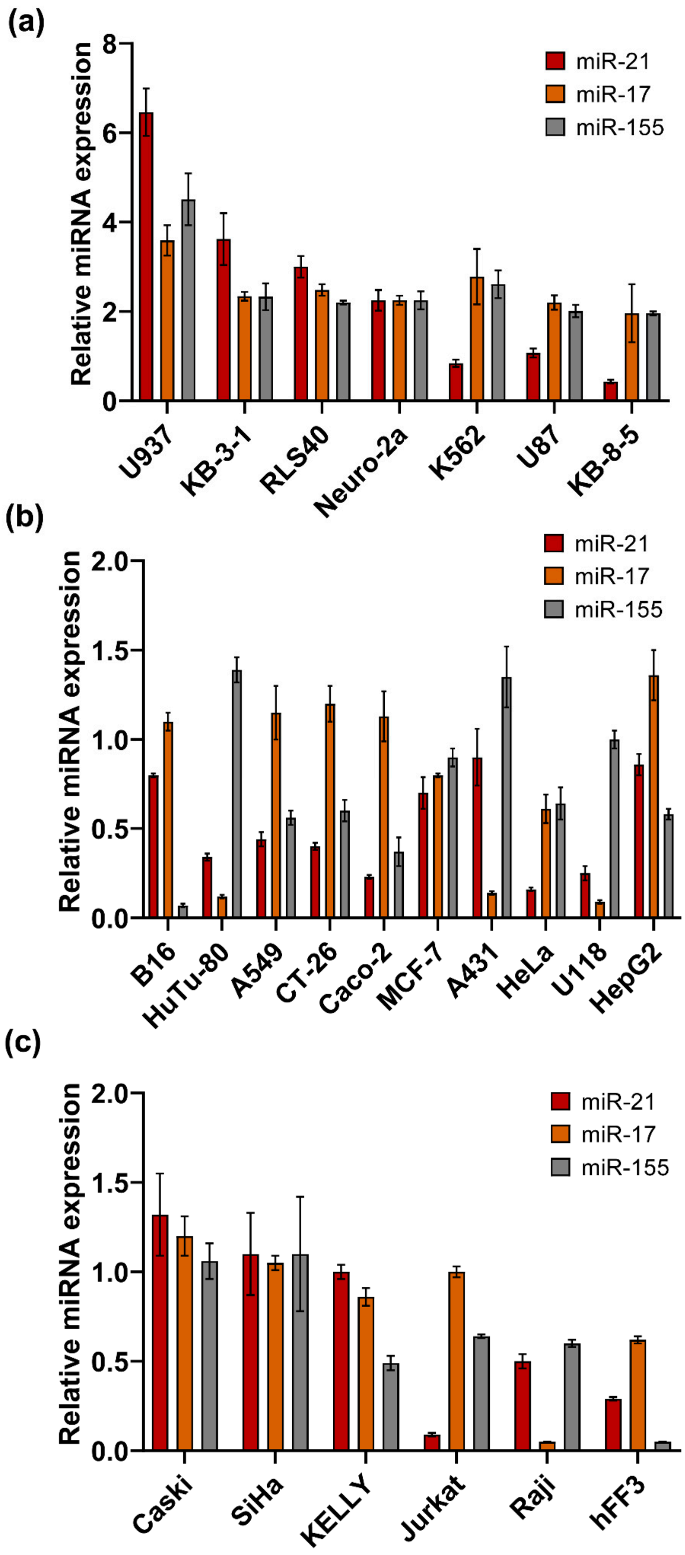


**Figure S3.** Basal expression of miR-21, miR-17 and miR-155 in all studied cell lines. (**a**) Cell lines with high basal levels of studied miRNAs. (**b**, **c**) Cell lines with lower basal expression of miRNAs exhibiting high to moderate (**b**) or low-to absent sensitivity (**c**) to μ-AMOs targeted to miR-21, miR-17, and miR-155. Results of stem-loop RT-PCR. The level of miRNAs was normalized to the level of small nuclear RNA U6.
